# Selective oxytocin receptor activation prevents prefrontal circuit dysfunction and social behavioral alterations in response to chronic prefrontal cortex activation in male rats

**DOI:** 10.3389/fncel.2023.1286552

**Published:** 2023-12-07

**Authors:** Philipp Janz, Frederic Knoflach, Konrad Bleicher, Sara Belli, Barbara Biemans, Patrick Schnider, Martin Ebeling, Christophe Grundschober, Madhurima Benekareddy

**Affiliations:** ^1^Roche Pharma Research and Early Development, Neuroscience and Rare Diseases Discovery and Translational Area, Roche Innovation Center Basel, F. Hoffmann-La Roche AG, Basel, Switzerland; ^2^Roche Pharma Research and Early Development, Therapeutic Modalities, Roche Innovation Center Basel, F. Hoffmann-La Roche AG, Basel, Switzerland; ^3^Roche Pharma Research and Early Development, Pharmaceutical Science, Roche Innovation Center Basel, F. Hoffmann-La Roche AG, Basel, Switzerland; ^4^Calico Life Sciences, South San Francisco, CA, United States

**Keywords:** prefrontal, social, oxytocin, chemogenetic, pharmacology, electrophysiology

## Abstract

**Introduction:**

Social behavioral changes are a hallmark of several neurodevelopmental and neuropsychiatric conditions, nevertheless the underlying neural substrates of such dysfunction remain poorly understood. Building evidence points to the prefrontal cortex (PFC) as one of the key brain regions that orchestrates social behavior. We used this concept with the aim to develop a translational rat model of social-circuit dysfunction, the chronic PFC activation model (CPA).

**Methods:**

Chemogenetic designer receptor hM3Dq was used to induce chronic activation of the PFC over 10 days, and the behavioral and electrophysiological signatures of prolonged PFC hyperactivity were evaluated. To test the sensitivity of this model to pharmacological interventions on longer timescales, and validate its translational potential, the rats were treated with our novel highly selective oxytocin receptor (OXTR) agonist RO6958375, which is not activating the related vasopressin V1a receptor.

**Results:**

CPA rats showed reduced sociability in the three-chamber sociability test, and a concomitant decrease in neuronal excitability and synaptic transmission within the PFC as measured by electrophysiological recordings in acute slice preparation. Sub-chronic treatment with a low dose of the novel OXTR agonist following CPA interferes with the emergence of PFC circuit dysfunction, abnormal social behavior and specific transcriptomic changes.

**Discussion:**

These results demonstrate that sustained PFC hyperactivity modifies circuit characteristics and social behaviors in ways that can be modulated by selective OXTR activation and that this model may be used to understand the circuit recruitment of prosocial therapies in drug discovery.

## Introduction

Social impairment is a transdiagnostic hallmark of different neurodevelopmental and neuropsychiatric conditions such as autism spectrum disorders (ASD) and schizophrenia (SZ). In a healthy brain, social behavior is tightly controlled by the PFC, which integrates sensory information to create mental representations that influence social decision-making and actions [reviewed in Forbes and Grafman (2010), Yizhar and Levy, 2021)]. Given its intricate connectivity not only with cortical regions involved in sensory processing and motor control, but also with subcortical brain areas of the limbic system (Ongür and Price, 2000; Wise, 2008), such as the nucleus accumbens, ventral tegmental area (VTA), lateral habenula (LHb) or the amygdala, the PFC is ideally placed to elicit top-down influence on attention, motivation and affect, which is highly-relevant for social behavior. In ASD and SZ, converging evidence from neuroimaging, physiological and systems neuroscience point to PFC dysfunction (Abdi and Sharma, 2004; Barak and Feng, 2016; Mubarik and Tohid, 2016) that may represent a common substrate for disease-related social deficits. On the mechanistic level, the disbalance of PFC excitation-to-inhibition has been extensively studied and shapes our current understanding of how complex genetics and environmental factors contribute to social deficits in psychiatric disorders (Bicks et al., 2015; Foss-Feig et al., 2017; Ferguson and Gao, 2018). Particularly the impairment of cortical interneurons appears as a central feature in both SZ (Lewis et al., 2005) and ASD (Contractor et al., 2021). In fact, several mechanistic rodent studies impressively showed that shifting excitation-to-inhibition balance in the PFC e.g., by silencing interneurons (Yizhar et al., 2011) or by exciting pyramidal neurons (Benekareddy et al., 2018) disrupts socially-directed behavior.

From a therapeutic perspective, several pharmacological approaches are being explored that target social brain circuits, with the aim to alleviate disease-associated social deficits. One of these approaches relies on the application of oxytocin, a neuropeptide that is particularly well-known for its role in modulating social behavior [reviewed in Meyer-Lindenberg et al. (2011), Stoop (2012), Young and Barrett (2015)] and anxiety (Triana-Del Río et al., 2019). However, despite promising preclinical results (Meziane et al., 2015; Peñagarikano et al., 2015; Hara et al., 2017; Kitagawa et al., 2021; Choe et al., 2022), and meta-studies arguing for an improvement of social functioning in ASD with intranasal oxytocin (Huang et al., 2021), recent studies performed in children with autism showed no improvement of social and cognitive function (Bernaerts et al., 2020; Sikich et al., 2021; Guastella et al., 2023). These discrepancies demonstrate the need to better understand how oxytocin affects brain circuits governing social behavior. In the mammalian brain the major sources of oxytocin are hypothalamic neurons projecting to widely distributed brain areas, including the PFC (Knobloch et al., 2012). Oxytocin that is released upon social cues may, beside its well-known neuroendocrine effects (Brownstein et al., 1980), have a wide-spread influence on brain networks. In the PFC, oxytocin activates interneurons that, in turn, inhibit either layer 2/3 pyramidal neurons in male mice to reduce anxiety, or layer 5 pyramidal neurons in female mice to elicit prosocial behavior (Li et al., 2016). However, in the PFC also pyramidal cells express OXTR, and optogenetic overactivation of these cells lead to deficits in social recognition (Tan et al., 2019). For the hippocampus it has been shown that oxytocin enhances signal transmission by increasing the activity of fast-spiking interneurons (Owen et al., 2013). Similarly, oxytocin boosts inhibition in the amygdala to attenuate fear responses (Knobloch et al., 2012). In the VTA oxytocin increases action potential firing of nucleus accumbens-projecting dopaminergic neurons to gate social reward (Hung et al., 2017). Treatment with oxytocin has been shown to rescue social deficits in a variety of rodent models relevant for ASD (Meziane et al., 2015; Peñagarikano et al., 2015; Hara et al., 2017; Kitagawa et al., 2021; Choe et al., 2022). However, from these studies it remains unclear if oxytocin can restore social behavior by acting specifically on OXTR in prefrontal circuits given its short half-life and its pharmacological effect on the vasopressin1a receptor (V1aR).

Therefore, in our study we developed a PFC circuit model for chronic social impairment, explored the underlying cellular mechanisms and tested its utility for drug development. In this context, we describe for the first time a selective, brain-penetrant peptidic OXTR agonist that was capable of preventing social-circuit dysfunction observed in our model.

## Materials and methods

### Animals

Adult male Sprague Dawley rats (Charles River, Saint-Germain-sur-l’Arbresle, FR), with an approximate weight of 250g at the time of arrival, were maintained on a reversed 12-hour light/dark cycle with food and water *ad libitum*. Experiments were conducted in adherence to the Swiss federal ordinance on animal protection. The use of male rats was motivated by epidemiological findings showing that the prevalence of ASD diagnosis is estimated to be around 3- to 4-fold higher in males compared to females (Loomes et al., 2017; Posserud et al., 2021).

### Viral injections

Rats were deeply anesthetized using a balanced anesthesia protocol (fentanyl 0.005 mg/kg + medetomidine 0.15 mg/kg + midazolam 2 mg/kg). Adeno-associated viruses (AAV) packaged by the Viral Vector Facility of the ETH Zürich were injected stereotaxically into the medial PFC (coordinates relative to bregma: Anterior-posterior, AP = + 2.73 mm; medio-lateral, ML = ± 0.6 mm; dorso-ventral, DV = −4.5 mm; 900 nL of AAV8-mCaMKIIa-hM3D(Gq)_mCherry-WPRE-hGHp) to express the depolarizing designer receptor exclusively activated by designer drug (DREADD) hM3Dq. For control rats, a control virus carrying only the genomic sequence for the fluorophore, but not for the DREADD, was used. In a subset of rats a retrograde-infecting AAV was additionally injected in the dorsomedial thalamus (coordinates: AP = + 3.20 mm, ML = ± 0.65 mm, DV = −5.8 mm; 150 nL of AAV-retro-CAG-EGFP-WPRE-SV40p(A)) to label a subpopulation of PFC projection neurons for subsequent whole-cell recordings.

### Peptide synthesis

RO6958375 was synthesized via microwave-based solid phase Fmoc-chemistry using the Liberty Lite system (CEM, Matthews, US). Synthesis scale was executed at 0.25 mmol with coupling times of 5 minutes per amino acid at elevated temperature (78°C) applied. The synthesis was carried out using TentalGel-S RAM resin as a solid support (0.24 meq/g). All amino acids used were dissolved in DMF to a 0.2 mol/L concentration. A mixture of HOBT/HBTU 1:1 (0.5 mol/L, 4 eq.) and DIPEA (4 eq.) was used to activate the amino acids. Fmoc-cleavage was achieved with piperidine in DMF (20%) for 3 min. The Fmoc-cleavage step was repeated. For Alloc- and allyl-cleavage, the resin was treated manually with a solution of 20 eq. of phenylsilane and 0.05 eq. of tetrakis(triphenylphosphine)palladium(0) in DCM (5 ml) for 30 min at RT. This procedure was repeated. The resin was washed with a solution of 0.5% sodium dithiocarbamate in DMF twice. The washing step was repeated with DCM. For on-bead cyclization, the coupling-reagent (4 ml of an 0.5 mol/L solution HOBT/HBTU (1:1) and 1 ml of DIPEA (4 eq.) in DMF was added to the resin. The slurry was shaken for 8 h at RT. The resin was washed with DMF and DCM twice. Completion of cyclization was verified via ninhydrin test. To cleave the peptide from the resin, 10 ml of the cleavage-cocktail (TFA/TIS/water, ratio of: 95/2.5/2.5) was added to the resin, and the mixture shaken at RT for 1h. Cleaved peptide was precipitated from cold ether (−18°C). The peptide was centrifuged, and the precipitates were washed twice with cold ether. The precipitate was dissolved in water/acetonitrile and lyophilized. Finally, the crude peptide was purified by preparative HPLC on a Reprospher 100 C18-T Column (100 × 4.6 mm, Sum particle size). As an eluent system a mixture of 0.1% TFA/water/acetonitrile was used with a gradient of 0–50% acetonitrile within 0–30 min. The fractions were collected and checked by analytical HPLC. Fractions containing pure product were combined and lyophilized. 7.2 mg of white powder was obtained. The product was analyzed via Electrospray Mass Spectrometry with a mass observed of 989.3 (M + H + : expected 989.1).

### Pharmacological characterization of OXTR agonist RO6958375

The peptidic OXTR agonist RO6958375 was profiled in a calcium release *in vitro* assay on cells expressing human or rat OXTR or the related human or rat vasopressin V1a, V1b and V2 receptors as previously described (Schnider et al., 2020). In a variation to the published protocol, cells were plated for 24 h at 50,000 cells/well in clear-bottomed 96-well plates, washed and dye-loaded for 2 hours with FLIPR calcium 6 no wash assay kit (Molecular Device) in assay buffer (1x Hanks Balanced Salt Solution, 20 mM Hepes) without adding probenicid. The plates were loaded on a fluorometric imaging plate reader (FLIPR) to measure calcium flux after addition of agonist.

### Peptide *in vivo* distribution studies

Adult female or male Wistar rats weighing circa 250 g were obtained from Harlan Laboratories (Horst, The Netherlands) and housed in a controlled environment (temperature, humidity, and 12-h light/dark cycle) with access to food and water *ad libitum*. All rodent studies were conducted with the approval of the local veterinary authority in adherence to the Swiss federal regulations on animal protection and to the rules of the Association for Assessment and Accreditation of Laboratory Animal Care International (AAALAC). Rats were administered RO6958375 subcutaneously at 0.03, 0.1, 0.3, or 4 mg/kg to cover a large range of exposure levels. Serial blood and one CSF sample were collected from each animal under deep anesthesia with 5% isoflurane in pure oxygen. Due to the expected very rapid apparent elimination kinetic, blood sample collection was performed from 0.25 to 4.5 h post-dose, with CSF sampling from 0.8 to 4.5 h. Blood samples were collected by heart puncture into K2EDTA coated polypropylene tubes and placed on ice. Plasma was prepared within 30 min by centrifugation at 3000 g for 5 min at 4°C and frozen immediately. Between 50 and 100 uL of CSF were collected by cisternal puncture of the atlanto-occipital membrane with a 22-gauge needle through silastic tubing in a 96- well plate. All samples were stored at 20°C. Compound concentrations in plasma and CSF were determined by means of LC–MS/MS. Merged PK data were analyzed by non-compartmental analysis and PK modeling (details not shown).

### Pharmacological treatment

Two weeks after surgery, the hM3Dq ligand clozapine-*N*-oxide (CNO; BML-NS105, Enzo Life Sciences, Farmingdale, USA) was injected at a dose of 1 mg/kg s.c. on a daily basis for 10 days, followed by a wash-out phase of 7 days. For the RO6958375 experiment CNO was provided in drinking water with a calculated final dose of 2 mg/kg. To test the effect of pharmacological intervention, the peptidic OXTR agonist RO6958375 (or a vehicle control) was injected once daily during the CNO wash-out phase (7 days in total). The dose was 0.07 mg/kg (s.c.) if not noted otherwise.

### Three-chamber sociability test

Rats (∼350g) were placed in a 3-chamber task from Noldus Ethovision (Noldus, Wageningen, NL). At each end of the rectangular box are the enclosures for the social stimulus (unfamiliar, age/weight-matched conspecifics of the same sex) or the non-social stimulus (unfamiliar object). At the beginning of the test, the rat is placed in the center chamber and allowed to explore the box for 10 mins (habituation). After that, a stimulus rat from a different cage is placed in one of the enclosures and the unfamiliar object in the opposing enclosure. The test rat is allowed to explore the environment for 10 mins (test phase). At the end of 20 minutes, the test rat and stimulus rat were taken out of the arena and the arena was cleaned with 30% alcohol before the next trial. Video was recorded, tracked and analyzed using Ethovision. The light level in the box is maintained at 20 Lux with indirect lighting. Data are expressed as sociability index = 100 × (time in social interaction/total interaction time) - 50. A social interaction is counted when the rat is in direct proximity to the enclosure with the stimulus rat.

### Electrophysiology

For terminal experiments rats were deeply anesthetized with 2.0% isoflurane (Abbott, Cham, CH) and decapitated. The brain was quickly removed and transferred to the slicing chamber filled with ice-cold N*-*methyl-D-glucamine (NMDG) solution containing (in mM) 110 NMDG, 3 KCl, 1.1 NaH_2_PO_4_, 25 NaHCO_3_, 103.02 HCl, 25 D-glucose, 10 *L*-ascorbic acid, 3 pyruvic acid, 0.5 CaCl_2_ * 2H_2_O and 10 MgCl_2_ * 6H_2_O. The brain was chopped into 350 μm thick frontal sections using a VT1000S vibratome (Leica, Wetzlar, GER). Acute slices were recovered in NMDG solution for 15 min at 35°C and then transferred either to normal ACSF containing (in mM) 124 NaCl, 2.5 KCl, 1.25 KH_2_PO_4_, 26 NaCO_3_, 10 *D*-glucose, 4 sucrose, 2 MgSO_4_ * 7H_2_O and 2.5 CaCl_2_ * 2H_2_O for extracellular recordings, or to ACSF optimized for whole-cell recordings containing (in mM) 124 NaCl, 2.5 KCl, 1 NaH_2_PO_4_, 25 NaHCO_3_, 20 *D-*glucose, 2 CaCl_2_ * 2H_2_O and 1 MgCl_2_ * 6H_2_O. Both solutions were adjusted to 305–310 mOsm. Patch-clamp whole-cell recordings were performed at RT and a perfusion rate of 1.5 ml/min, whereas spontaneous action potentials were recorded at about 35°C and a perfusion rate of 3 ml/min.

For testing synaptic transmission within the PFC a bipolar stimulation electrode was located in layer 2/3 and a borosilicate pipette (GC150F-10; Harvard Apparatus, Cambridge, USA) filled with a solution of (in mM) 150 NaCl, 3.5 KCl, 10 HEPES, 10 *D-*glucose, 1.3 MgCl_2_ * 6H_2_O, 2.5 CaCl_2_ * 2H_2_O and a resistance of 2.5–3.5 MΩ was placed in layer 5 to record evoked potentials. The recording electrode was lowered 150 μm into the tissue to obtain maximal responses. Stimulation currents were generated with an STG3000 and MC Stimulus II software (Multi Channel Systems, Reutlingen, Germany). The signal was preamplified with a CV-7B electrode holder (Axon Instruments, Molecular Devices, San Jose, California, US) and again amplified and digitized with a MultiClamp 700B (Axon Instruments) using 100xAC membrane potential (200 mV/mV) mode, a bessel filter at 2.4 kHz and alternating current at 1 Hz. Signals were recorded with Clampex software (Axon Instruments). First, an input-output protocol was performed by increasing the stimulation intensity till 300 μA in 20 μA current steps. For electrical pulses we used a square shape and a duration of 100 μs. Then, we performed a paired pulse protocol using intervals of 20, 50, 100, 200, 400 and 800 ms and a stimulation current (typically 80–100 μA) which elicited the half-maximum slope of the postsynaptic field potential (fPSP). Finally, synaptic short term depression (STD) was tested by delivering 40 pulses at 5, 10, 20 and 40 Hz. All protocols were performed in 3 slices for each rat and repeated three times. The amplitudes and slopes of the presynaptic fiber volley (FV) and the fPSP were semi-automatically analyzed using Clampex and averaged per animal.

Spontaneous activity was recorded in the central LHb and the VTA using the same pipette solution and amplifier settings as mentioned above. Active neurons were identified by slowly protruding the electrode forward into the tissue. As soon as recurrent spikes occurred in the voltage trace the electrode position was fine-adjusted to obtain a signal amplitude between 0.2 and 0.5 mV. After waiting 5 min for the spike rate to normalize, action potential firing was recorded for at least 10 min. The firing frequency was analyzed for each neuron by using an adjustable threshold for spike detection in Clampex. Spike shapes were checked for each recording to ensure that the calculated firing frequencies relate to individual neurons.

For patch-clamp experiments PFC layer 5 pyramidal neurons were morphologically identified and recorded in whole-cell mode. The patch pipette (GC150F-10; Harvard Apparatus) contained a solution of (in mM) 145 KMeSO_4_, 10 Hepes, 10 NaCl, 10 EGTA, 5 MgATP, 0.5 Na_2_ATP, 1 CaCl_2_ * 2H_2_O and 1 MgCl_2_ * 6H_2_O (adjusted to 290 mOsm and pH 7.2) and had a resistance 2.5–3.5 MΩ. Using positive pressure neurons were visually approached. By applying negative pressure a gigaseal was formed with the targeted neuron. Then, the cell membrane was opened and protocols were started 10 min after opening, to allow for sufficient exchange of intracellular solution and normalization of cell physiology. Cell viability was tested before and after each trial. The membrane potential was held at −70 mV, and only neurons for which leak currents did not exceed ± 100 pA and for which the axial resistance did not show changes higher than 20% were included for analysis. After inferring the basic neuronal properties (i.e., the membrane resistance, R_*m*_; the membrane capacity, C_*m*_; and the resting membrane potential, V_*rest*_), steady-state potassium currents were assessed by holding the membrane potential at −40 or 0 mV for 500 ms respectively. Intrinsic excitability was inferred by quantifying the number of evoked generated action potentials during incremental current steps of 20 pA (100 ms duration) until a stimulus intensity of 500 pA was reached.

Of note, acute slice experiments for all cohorts were done over several days, with one experiment/rat per day for each cohort (cohort used for characterization: 8 rats in total = 8 test days, timeframe of experiment: days 8–18 after last CNO dosing; cohort used for RO6958375 experiments: 12 rats in total separated in two cohorts = 6 test days, timeframe of experiment: days 8–16 after last CNO dose).

### RNA sequencing

The mPFC was dissected out from the sections after electrophysiology, and the tissue was flash frozen in liquid nitrogen and stored at −80°C until further use. RNA was extracted using Qiagen RNeasy mini kit (Qiagen, Hilden, GER). RNA quality was checked using Agilent RNA 6000 Nano Kit (Agilent Technologies, Santa Clara, US), RNA concentration was measured with Nanodrop (Thermo Fisher Scientific, Waltham, US). 500 ng of RNA was used as input for library preparation using the Illumina TruSeq Stranded mRNA library prep kit (Illumina, San Diego, US) according to the manufacturer’s instructions. Libraries were quality checked on a Fragment Analyzer (Agilent Technologies) and concentration was measured with Qubit dsDNA HS Assay kit (Thermo Fisher Scientific). Libraries were pooled and sequenced on a HiSeq4000 (Illumina).

### Statistical analysis

Behavioral and electrophysiological data was tested statistically with Prism 7 software (GraphPad Software Inc., La Jolla, USA). Applied statistical tests are described in the figure captions. Numeric values and statistical tests for key results are provided in Supplementary Table 1. Transcriptome data was analyzed using one-way ANOVA followed by a *post hoc* test corrected for multiple comparisons using the original FDR method (Benjamini and Hochberg, 1995).

## Results

### CPA impairs socially-directed behavior

**FIGURE 1:**
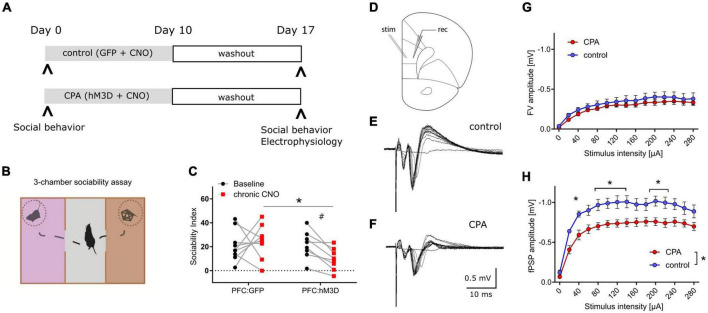
Chronic prefrontal activation impairs social behavior and PFC synaptic transmission. **(A)** Schematic of experiment to induce prolonged activation of the prefrontal cortex using a combination of hM3Dq expression in the excitatory neurons and repeated administration of the ligand CNO. **(B)** Cartoon representation of the 3-chamber sociability task used to measure social preferences. **(C)** Sociability index measurements after chronic PFC activation. The control group was transduced with AAV:GFP while the treatment group was transduced with AAV:hM3Dq. Social behavior was measured before and after chronic CNO treatment. ^#^*p* = 0.066 using two-way ANOVA followed by Fisher’s LSD test. Group comparison was done using Sidak’s *post hoc* test *<0.05. **(D)** Schematic of experimental design: Extracellular stimulation was performed in PFC layer 2/3 and evoked responses were recorded in PFC layer 5 of acute slice preparations. **(E,F)** Representative evoked responses under control and chronic prefrontal activation (CPA) conditions. Note that for individual current steps (0–280 uA) resulting voltage traces are overlaid. **(G,H)** Quantitative analysis of two components of evoked responses (fiber volley, FV; postsynaptic field potential, fPSP) reveals a significant reduction in fPSP amplitudes for the CPA group. Data is displayed as mean ± SEM. Two-way ANOVA with Sidak‘s *post hoc* test was performed for statistical comparison. To compare the overall difference between control and CPA conditions, respective AUCs were tested with Student‘s *t*-test. *p < 0.05.

To increase the neuronal activity of the prefrontal network over many days, hM3Dq was expressed in PFC pyramidal neurons, and repeatedly activated over 10 days (Figure 1A). Social behavior was assessed at baseline and one week after the phase of prefrontal hyperactivity using the three-chamber sociability task (Figure 1B). We found that CPA rats, whose PFC was repeatedly activated, showed a strong trend toward fewer interactions with an unfamiliar conspecific (Figure 1C). Control rats that were transduced only with the control fluorophore, exhibited no group level decrease in social interaction. Comparison between cohorts revealed that the sociability index is significantly decreased following CPA compared to controls (Figure 1C), while locomotor activity was not affected (Supplementary Figure 5A). We observed test-retest variability of the SI in the PFC:GFP group which was non-significant at a group level but highlighted the variability of this ‘naturalistic behavior’.

### Prefrontal synaptic transmission is disrupted subsequent to CPA

Having revealed a chronic impairment of social behavior upon CPA, we aimed to elucidate the underlying cellular and circuit changes. First, we investigated synaptic transmission within the PFC in acute slices in which cells in layer 2/3 were stimulated with a bipolar electrode, while evoked potentials were recorded in layer 5 (Figure 1D–F). Remarkably, the amplitudes of evoked field potentials were significantly decreased in CPA rats compared to controls (Figure 1H). Fiber volley amplitudes showed a similar trend (Figure 1G). To assess whether the decrease in neuronal responses might be a result of changed inhibitory feedback or presynaptic release properties, we also performed paired-pulse experiments and short-term depression experiments (Supplementary Figure 1). Neither the paired-pulse ratios nor synaptic depression were different between the two experimental groups, showing that local inhibitory circuits and presynaptic plasticity mechanisms remain intact following CPA.

### CPA decreases excitability of prefrontal projection neurons

**FIGURE 2:**
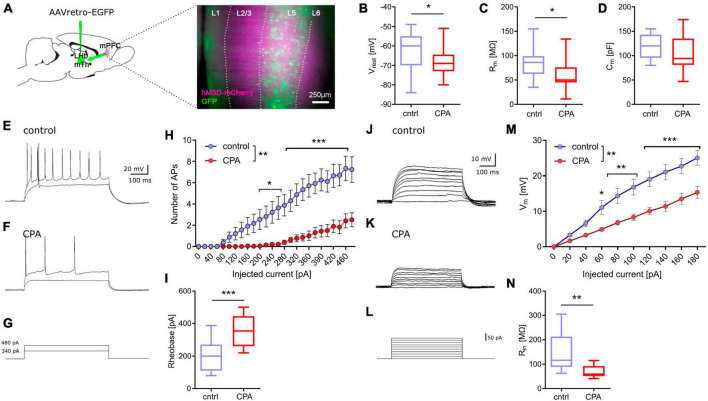
Chronic prefrontal activation reduces excitability of PFC output neurons. **(A)** Schematic for whole-cell recording of PFC output neurons: Infusion of a retro-AAV reporter in the region of the lateral habenula (LHb) and the mediodorsal thalamus (MTh) allowed to perform whole-cell recordings from retrogradely-labeled PFC projection neurons (green) also expressing hM3Dq-mCherry (magenta). **(B–D)** Quantitative analysis of membrane properties (resting membrane potential, Vrest; membrane resistance, Rm; membrane capacitance, Cm) indicate that PFC projection neurons are more hyperpolarized and show a lower membrane resistance following chronic hyperactivation. To test whether these intrinsic changes affect neuronal excitability, current injections were performed. **(E–G)** Representative voltage traces for 340 and 480 pA current steps. **(H)** Quantitative analysis of the number of resulting action potentials (APs) reveals that the intrinsic excitability is dramatically reduced under CPA conditions, resulting in panel **(I)** an increase in the rheobase. **(J–L)** Representative traces for subthreshold voltage steps and resulting membrane potential, and **(M)** corresponding quantification showing the decreased Input-output relation following CPA. **(N)** Input resistance calculated for 80 pA. Students‘s *t*-test was performed to test for statistical differences between groups. Two-way ANOVA with Sidak‘s *post hoc* test was performed for statistical comparison in panels **(H,M)**. *<0.05, **<0.01, ***<0.001. Data in box-and-whiskers plots is displayed as the median with min. to max. All other data is displayed as mean ± SEM (box).

**FIGURE 3:**
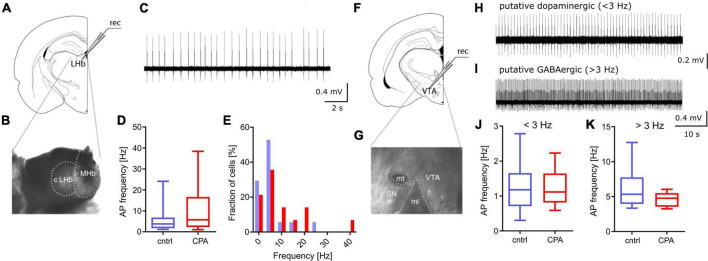
Spontaneous activity in PFC downstream targets LHb and VTA remains unchanged. **(A,B)** Schematic and representative microscopy image of the location for extracellular recording in the LHb. **(C)** Representative voltage trace from a spontaneously active neuron. **(D,E)** Respective quantitative analysis of the AP frequency and the distribution of spontaneously active cells, shows no significant difference between the control and CPA group. **(F,G)** Schematic and representative microscopy image of the location for extracellular recording in the VTA. **(H,I)** Representative voltage traces from a putative dopaminergic cell (slow spiking, <3 Hz) and a putative GABAergic (> 3 Hz spiking) neuron. **(J,K)** Quantitative analysis of AP frequencies for both cell populations shows no significant groups differences. Statistical comparison was performed with Student‘s *t*-test. Data is displayed as the median with min. to max (box-and-whiskers).

Next, we addressed putative changes on the single-cell level that could explain the impaired synaptic transmission by performing whole-cell patch-clamp recording of layer 5 pyramidal neurons (Figure 2). To be able to record specifically from PFC projection neurons, we labeled these cells by infusion of a retrograde-traveling reporter virus targeting the mediodorsal thalamus and habenula (Figure 2A). PFC projection neurons from CPA rats showed a significantly hyperpolarized resting membrane potential and reduced membrane resistance (Figures 2B, C), as well as reduced intrinsic excitability upon current injections with a nearly doubling of the rheobase (Figures 3H, I). In line with the reduced excitability, we found that subthreshold current injections in neurons from CPA rats resulted in smaller increases of the membrane voltage and decreased input resistance (Figures 2J–N). Since the membrane potential and input resistance is critically regulated by several types of potassium channels, we inferred steady-state potassium currents. In particular, voltage-gated potassium currents elicited at −40 mV were strongly elevated and, as expected, the magnitude of these currents positively correlated with the rheobase (Supplementary Figures 2C, D). Similar differences were also evident for potassium currents elicited with 0 mV holding potential (Supplementary Figures 2G, H).

### Spontaneous activity of PFC-downstream targets LHb and VTA remains intact following CPA

Going beyond the PFC, we studied the function of two key downstream targets relevant in social behavior, the LHb and the VTA (Figure 3). Considering that a large fraction of neurons within the LHb and VTA are spontaneously active, we performed extracellular recording of action potentials. Our results show that CPA does not lead to long-term changes of firing rates, in the LHb or the VTA (Figures 3D–K), suggesting that these PFC-downstream targets may not be affected by CPA.

### Pharmacological and pharmacokinetic characterization of the novel peptidic OXTR agonist RO6958375

**FIGURE 4:**
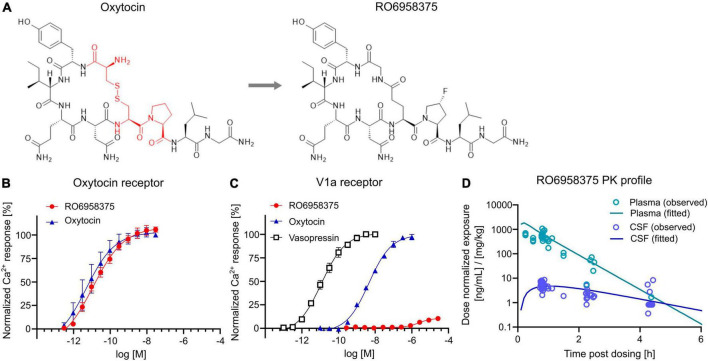
OXTR activation and pharmacokinetics of RO6958375. **(A)** Chemical structure of oxytocin (with the changed amino acids highlighted in red) and our novel selective OXTR agonist RO6958375. **(B,C)** Calcium release data of oxytocin and RO6958375 in rat OXTR and V1aR expression systems. **(D)** *In vivo* SC pharmacokinetics of RO6958375 in rat plasma (negligible plasma protein binding) and CSF from different studies. Data is displayed as mean ± SEM.

To assess the therapeutic potential of OXTR activation on social circuit impairment following CPA, we tested our novel selective OXTR agonist RO6958375. An extensive structure-activity relationship study of oxytocin revealed that substitution of the cysteine bridge by a lactam (Figure 4A) resulted in an unprecedented selectivity against vasopressin receptor 1a (V1aR). *In vitro* functional studies in a Gq-coupled Ca^2+^ release assay demonstrated that RO6958375 is a full OXTR agonist and equipotent to oxytocin on rat OXTR (Figure 4B), while it is inactive on rat V1aR up to 1 μM. In contrast, oxytocin fully activated rat V1aR at 1 μM compared to the endogenous full agonist vasopressin (Figure 4C). RO6958375 also fully activated the human OXTR and was inactive on human V1aR up to 27 μM (data not shown). EC_50_ values are provided in Supplementary Table 2. Furthermore, RO6958375 did not show any significant radioligand displacement or functional inhibition in a panel of 45 human receptors, channels, transporter and enzymes (see Supplementary Table 3).

For *in vivo* distribution studies the CNS uptake of RO6958375 after subcutaneous (SC) administration was tested in various single-dose pharmacokinetic studies in rats. Subcutaneous absorption was rapid, with a half-life in plasma of ca. 10–15 min. Interestingly, the CSF kinetic results indicated a longer residence of RO6958375 in brain compared to plasma (brain half-life of ca. 70 min) with a CNS uptake of ca. 1% from plasma (Figure 4D). From the above studies, the highest concentrations detected in rat CSF at ca. 0.8 h after single SC administration of 0.03 or 0.1 mg/kg was equivalent to 0.24 and 0.58 nM, which is more than 10-fold above the *in vitro* OXTR EC_50_. RO6958375 is therefore sufficient to fully activate the rat OXTR for 4.3 h post-dose. For *in vivo* studies we selected subcutaneous doses of 2.4*10E-8, 0.001 and 0.07 mg/kg, leading to 0, 10 or 72% brain OXTR activation respectively, based on the *in vitro* OXTR dose-response. Of note, we did not perform head-to-head comparison of the pharmacokinetic profile of RO6958375 and oxytocin. While RO6958375 is a synthetic compound which can be distinguished from endogenous oxytocin, CSF quantification of administered oxytocin may be confounded by endogenous oxytocin released in the brain (for example due to stress during injection).

### Sub-chronic selective OXTR activation prevents CPA-induced prefrontal circuit dysfunction

**FIGURE 5:**
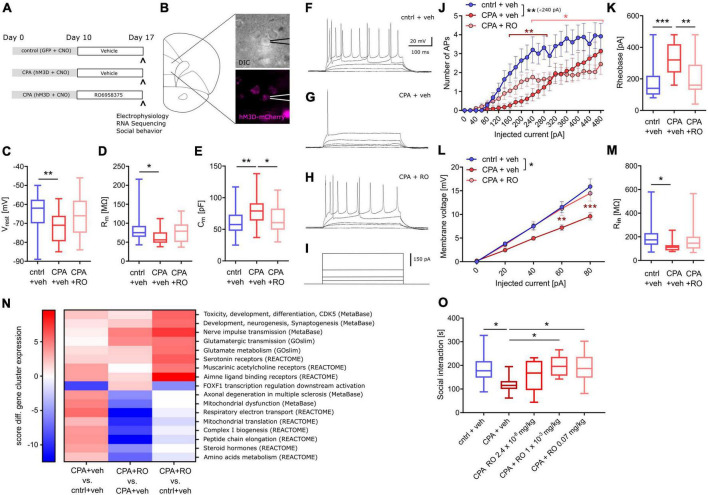
Chronic OXTR agonist treatment prevents circuit dysfunction following PFC overactivation. **(A)** Overview of experimental groups and treatment plan. **(B)** Schematic of the site of whole-cell recording from PFC layer 5 pyramidal neurons and a microscopy image of a patched cell expressing hM3Dq-mCherry (magenta). **(C–E)** Quantitative analysis of membrane properties (resting membrane potential, Vrest; membrane resistance, Rm; membrane capacitance, Cm) showing that intrinsic changes that occur upon prolonged prefrontal hyperactivity (CPA) in PFC pyramidal neurons are prevented or attenuated in the OXTR agonist-treated (RO6958375, RO) group. To test whether these intrinsic changes affect neuronal excitability, current injections were performed. **(F–I)** Representative voltage traces at 0, 20, 40, 340, and 480 pA current injections. **(J,K)** Quantitative analysis of the number of action potentials (APs) produced during each current step and the calculated rheobase (current needed to produce the first AP), reveals that the intrinsic excitability is dramatically reduced under CPA conditions and attenuated in the OXTR agonist-treated group. To probe whether the decreased excitability is associated with a reduced input resistance the current-voltage (IV) relationship was analyzed: **(L,M)** Quantitative analysis of the IV-curve and the calculated input resistance (Rin) at 80 pA, shows that following CPA neurons need more current to produce the same membrane voltage which is associated with a strongly reduced input resistance. Remarkably, this deficit is not detectable in the OXTR agonist-treated group. One-way ANOVA with Tukey‘s *post hoc* test was performed to test for statistical differences between groups for one condition. Two-way ANOVA with Sidak‘s *post-hoc* test was performed for statistical comparison in J and L, in which two conditions were regarded (statistical differences from *post hoc* tests are indicated in the corresponding color for the condition and refer to a difference to control values). In addition, to compare the overall difference between conditions, respective AUCs were tested with one-way ANOVA and Tukey‘s *post hoc* test (statistical difference is depicted at the side of the legend). *p < 0.05, **<0.01, ***p < 0.001. **(N)** Heat map showing gene clusters (annotated using gene sets from: MetaBase, GeneOntology, REACTOME, and the Roche Molecular Phenotyping collection). Every gene network is categorized, for each contrast, by a significance score (positive for up-regulation, negative for down-regulation). As a first pass, for every gene set, we use the maximum of all the absolute scores to sort them. We only include gene sets with at least 3 genes and not more than 500 genes contributing. Based on this, we continue analyses with gene sets scoring at least ± 5 in any one of the contrasts analyzed. **(O)** Social behavior data shows a significant decrease in social interaction time with chronic PFC activation, an effect not observed in chronic PFC activation rats treated with OXTR agonists at 0.001 or 0.07 mg/kg. Data was analyzed using one-way ANOVA followed by Fisher’s LSD *post hoc* test. *p < 0.05. Data in box-and-whiskers plots is displayed as the median with min. to max. All other data is displayed as mean ± SEM (box).

First, we tested whether OXTR activation can prevent the PFC dysfunction following CPA. Therefore, rats were injected once daily with our novel selective peptidic OXTR agonist RO6958375 (RO, 0.07 mg/kg) or the vehicle for seven days, starting directly after CPA (Figure 5A). Subsequent to the treatment, whole-cell recording of PFC layer 5 pyramidal neurons was performed (one rat per day; see section “Material and methods” for details) and tissue was preserved for later transcriptomic analysis. Remarkably, we found that the clear differences in passive membrane properties, evident between control rats and CPA rats, are not observed in RO6958375-treated CPA rats (Figures 5C–E). Again, we also probed the intrinsic excitability by applying incremental current injections and found that RO6958375 treatment also fully restored the rheobase (Figure 5K), whereas the number of evoked action potentials is only partially recovered for lower current steps (Figure 5J). Consistent with the RO6958375-mediated restoration of membrane properties and intrinsic excitability, we also found that both, the current-membrane voltage relationship and the input resistance were normalized (Figures 5L, M). Notably, CPA-induced hypoexcitability and its reversal after subchronic RO6958375 treatment was observed over several days, indicating stable changes of cellular function under both conditions (Supplementary Figure 3).

### Sub-chronic selective OXTR activation partially normalizes CPA-induced transcriptomic changes

To better understand the molecular effects of CPA and selective OXTR agonism, we performed transcriptome analysis of tissue we collected following acute slice electrophysiology. RNA-Seq revealed wide-range transcriptomic changes in CPA rats, which were partially prevented with RO6958375 treatment (Figure 5N). Pathway analysis showed that gene networks associated with mitochondrial function, peptide metabolism, steroid hormones, muscarinic and amine ligand binding receptors, as well as axonal degeneration and toxicity were upregulated following CPA, whereas genes associated with transcriptional regulation of FOXF1 downstream activation were downregulated (Figure 5N, first column). RO6958375 treatment normalized the expression of a subset of gene networks, involved in mitochondrial function, peptide metabolism, steroid hormones and axonal degeneration (Figure 5N third column, lower half). However, RO6958375 treatment also augmented expression in certain gene networks, involved in developmental processes, nerve pulse transmission, glutamatergic metabolism and transmission, as well as several neurotransmitter receptor classes (Figure 5N third column, lower half). Beyond gene network alterations, we observed transcriptional dysregulation for several potassium channels and associated regulatory elements as well as sodium channels, which may contribute to the CPA-induced neuronal hypoexcitability and that were prevented with RO6958375 (Supplementary Figure 4). Of note, RNASeq did not show significant alterations of OXTR expression in any condition (data not shown), which has important implications for the interpretation of our data.

### Sub-chronic selective OXTR activation alleviates CPA-induced deficits in social interaction

Finally, we addressed whether the RO6958375-mediated restoration of PFC physiology and transcription also manifest on the behavioral level using a separate group of animals. Social behavior was again evaluated by using the three-chamber sociability test after CPA with RO6958375 or vehicle treatment. Indeed, the social deficits observed in CPA rats were not evident following RO6958375 treatment at 0.001 or 0.07 mg/kg compared to the vehicle treatment (Figure 5O). Locomotor activity was similar between conditions (Supplementary Figure 5B). Our results demonstrate the prosocial effect of selective OXTR agonism in this circuit model of social behavior impairment, substantiating its translational value.

## Discussion

Modeling social impairment seen in neurodevelopmental and neuropsychiatric conditions preclinically remains a challenge owing to the largely idiopathic nature of these conditions. To address this problem, we adopted a two-step approach, by developing a mechanistic model (the CPA rat model) of social dysfunction and utilizing our novel OXTR-specific agonist to demonstrate efficacy. The CPA model is based on chronic perturbation of the PFC, a candidate node in the social brain network also disrupted in SZ and ASD (see introduction). Previous circuit studies have delineated various neural pathways necessary and sufficient for orchestrating social behavior on acute timescales (Bicks et al., 2015; Yizhar and Levy, 2021). However, the utility of these circuit models in drug discovery remains limited due to the acute nature of these manipulations and the lack of validated pharmacological intervention paradigms. In this context, our work provides a unique systems biology framework where chronic dysfunction of PFC social-circuits is modeled preclinically and can potentially be used to gain insights into the mechanism-of-action of novel therapeutic interventions.

Several lines of evidence suggest that PFC E/I imbalance represents a common circuit substrate for various causes of social impairment in ASD (Rubenstein and Merzenich, 2003; Nelson and Valakh, 2015). Clinical evidence comes from gene mutations affecting excitability and synaptic function in ASD (Voineagu et al., 2011). In fact, 50–70% of children with ASD display physiological abnormalities indicative of cortical hyperactivity (Lewine et al., 1999; Dichter et al., 2009). Elegant experimental work has demonstrated that acute PFC hyperactivity reversibly impedes social interactions in rodents (Yizhar et al., 2011; Benekareddy et al., 2018). We hypothesized that perturbation of PFC function will also impair social behavior on longer time scales. Therefore, we chose a chemogentic-based approach for stimulating PFC activity over several days. Our finding that prolonged hyperactivity of PFC pyramidal neurons results in persistent hypoexcitability has important implications for experiments involving repeated modulation of neuronal activity via hM3Dq. To our knowledge, this is the first study revealing that hM3Dq-mediated hyperactivity downscales neuronal excitability reminiscent of homeostatic plasticity. In homeostatic plasticity, increased activity levels lead to a reduction of synaptic efficacy or intrinsic excitability to maintain an internal set point, e.g., the firing frequency or membrane potential [reviewed in Turrigiano (2012)]. In a disease context, similar ‘adaptive’ changes are known from epilepsy, in which network hyperactivity leads to a downscaling of neuronal excitability, due to upregulation of potassium channels (e.g., Kir2.1, HCN and Kv1.1 channels). The resulting increased potassium leakage reduces the resting membrane potential and input resistance, which impairs action potential generation (Stegen et al., 2009, 2012; Young et al., 2009; Kirchheim et al., 2013). We propose that similar mechanisms are engaged upon CPA, in which the hypoexcitability is associated with decreased resting membrane potential, input resistance and elevated potassium currents. Our transcriptome results corroborate this notion, showing that several genes encoding for potassium channels, associated regulatory elements and other ion channels are dysregulated following CPA. While assessing the specific role of individual transcriptional alterations was not in the scope of the present study, and our bulk transcriptomic approach does not delineate cell type-specific effects, our results point to complex ion channel alterations that may underlie CPA-mediated hypoexcitability. Importantly, gene network alterations involved in mitochondrial function and metabolism may also contribute to hypoexcitability, considering for instance that deficits in energy production will impair neuronal function. While the functional consequences on the network level were not addressed in the current study, it is tempting to speculate that CPA disrupts processing of afferent signals (e.g., social cues) in the PFC, which in turn impedes socially-directed behavior. Since OXTR expression remained unchanged in all experimental conditions, social dysfunction in the CPA model is unlikely a consequence of OXTR downregulation. Notably, our experiments showed that in the LHb and the VTA (Beier et al., 2017; Murugan et al., 2017; Benekareddy et al., 2018) spontaneous activity was not affected by CPA, which suggests that CPA impairs social behavior predominantly by disrupting PFC physiology, and not necessarily its downstream targets. This clearly differentiates the CPA model from the acute model, in which social impairment due to hM3Dq-mediated PFC overexcitation could be completely restored by inhibiting the LHb (Benekareddy et al., 2018).

Intriguingly, our electrophysiological data demonstrated that repeated OXTR activation prevented or reversed CPA-induced dysfunction of PFC pyramidal neurons. A sub-population of excitatory PFC neurons express OXTRs (Tan et al., 2019) and OXTR-activation has been shown to depolarize subicular neurons by activating TRPV1 channel activation of potassium-channel depression (Hu et al., 2021). In this context, it is reasonable to speculate that OXTR-activation engages similar direct mechanisms in the PFC to counteract CPA-induced hypoexcitability. Another plausible, though indirect, mechanism-of-action relies on neuronal inhibition. In line with the concept of homeostatic plasticity, enhanced inhibition could counteract the hyperactivity-induced downscaling of excitability by adjusting the internal set point of affected neurons. Indeed, oxytocin has been shown to boost inhibition in several brain areas, including the PFC (Li et al., 2016), the amygdala (Viviani et al., 2011; Knobloch et al., 2012), the auditory cortex (Marlin et al., 2015) and other sensory systems [reviewed in Grinevich and Stoop (2018)]. Recently, it has been demonstrated that early-life oxytocin treatment can rescue social deficits in a bilateral whisker trimming model and in Fmr1 KO mice by counteracting hippocampal hyperactivity (Pan et al., 2022). In the PFC, OXTRs are expressed by a subset of GABAergic interneurons (Nakajima et al., 2014) that could be activated via OXTR-signaling [reviewed in Stoop (2012), Jurek and Neumann, 2018]. Indeed, optogenetic activation of these interneurons leads to inhibition of PFC pyramidal neurons (Li et al., 2016). We suggest that this inhibition drives the internal set point of hyperactive PFC neurons away from their pathological state to restore PFC function and social behavior.

So far, studying the therapeutic effect of oxytocin has been challenging due to the short half-life, poor brain penetration and the inherent exposure variability associated with administration methods such as intranasal delivery. Therefore, the question remains whether enough oxytocin reaches the brain after peripheral administration to exert behavioral effects (Leng et al., 2022). Moreover, the interpretation of behavioral effects seen in studies using oxytocin may be hampered due to its agonistic activity on V1aR, which is of particular interest, since in adult rats oxytocin has been shown to elicit larger prosocial effects compared to the more OXTR-selective oxytocin-derivative TGOT (Suraev et al., 2014). To overcome these limitations we developed RO6958375, a derivative of oxytocin that shows outstanding selectivity for OXTR and sustained brain exposure. Our results show that a low dose of 0.001 mg/kg RO6958375, resulting in an estimated 10% brain OXTR receptor activation, is sufficient to elicit prosocial effects in our rodent model. In contrast, previous *in vivo* studies (Peñagarikano et al., 2015; Peñagarikano, 2017) typically use high oxytocin doses (1 mg/kg i.p.), which could result in OXTR-desensitization and peripheral V1aR activation, causing vasoconstriction that may interfere with behavior readouts. Compared to bivalent oxytocin agonists described previously (Busnelli et al., 2016), RO6958375 has the advantage of being smaller and therefore having a better brain penetration. An open question in the field concerns the right time for oxytocin-treatment in neurodevelopmental disorders, with many advocating for a treatment as early as possible. From our data, indeed, it remains unclear if OXTR activation is only efficacious in a certain time window during which CPA-driven alterations emerge or if treatment could even restore PFC physiology after chronification. Further studies are needed to better understand the mechanism-of-action and the disease-context for which OXTR activation will be therapeutically useful.

Oxytocin-release in the brain is well known to regulate several aspects of social behavior through a distributed network of OXTR-containing neurons, which receive oxytocinergic input mainly from the paraventricular nucleus [reviewed in Jurek and Neumann (2018)]. Studies in rodents showed that the PFC, the VTA, the amygdala and the nucleus accumbens are important network nodes to mediate acute prosocial and anxiolytic effects of oxytocin (Knobloch et al., 2012; Dölen et al., 2013; Song et al., 2016; Hung et al., 2017). In fact, it has been hypothesized that this may be specifically mediated by oxytocin acting on OXTRs (Song et al., 2016; Zhu et al., 2021). These studies can explain the acute prosocial effects of oxytocin on the behavioral level, as shown previously by others and by us in the current study. In addition, our findings on the electrophysiological properties and transcriptomic alterations expand this view, by speaking to a long-lasting restoration of PFC function by subchronic OXTR-application. Oxytocin has also been shown to have long-lasting effects on fear and social anxiety [reviewed in Triana-Del Río et al. (2019)], which could be mediated by plasticity-related gene expression downstream of OXTR-signaling [reviewed in Jurek and Neumann (2018)]. However, it is important to note that chronic OXTR-activation in a “healthy” brain may also be detrimental (Winter et al., 2021). Further investigations into the effect of OXTR-signaling on longer timescales would help to elucidate the therapeutic potential of OXTR agonists for the treatment of behavioral deficits in neurodevelopmental and neuropsychiatric disorders. Moreover, our work should motivate future studies, to assess in more depth the pharmacological consequences on social behavior and other behavioral domains as well as on the processing of relevant sensory stimuli. Given that the current study only tested the behavior in the 3-chamber sociability assay, we cannot exclude that the observed differences in social behavior may be due to increased levels of anxiety, deficits in olfactory sensation or changes in other relevant domains.

In conclusion, our study indicates that hyperactivity-dependent maladaptation could be a critical component in social circuit dysfunction relevant for ASD and SZ (Rubenstein and Merzenich, 2003; Kehrer et al., 2008; Markram and Markram, 2010; Vattikuti and Chow, 2010), and that selective OXTR activation could prevent the chronification of such deficits. Of note, as we only used male rats in the current study and taking into account the sex-specific regulation of the oxytocin system (Dumais and Veenema, 2016; Caldwell, 2018), our interpretations cannot not be extrapolated without caution to female rats.

## Data availability statement

The data presented in this study are deposited in the GEO (Gene Expression Omnibus, NCBI) repository, accession number GSE246666.

## Ethics statement

The animal study was approved by the Federal Food Safety and Veterinary Office of Switzerland. The study was conducted in accordance with the local legislation and institutional requirements.

## Author contributions

PJ: Conceptualization, Data curation, Formal analysis, Methodology, Visualization, Writing – original draft. FK: Conceptualization, Methodology, Supervision, Writing – review and editing. KB: Methodology, Visualization, Writing – review and editing. SB: Data curation, Formal analysis, Visualization, Writing – review and editing. BB: Methodology, Writing – review and editing. PS: Conceptualization, Writing – review and editing. ME: Formal analysis, Methodology, Writing – review and editing. CG: Conceptualization, Methodology, Project administration, Supervision, Writing – review and editing. MB: Conceptualization, Methodology, Project administration, Supervision, Visualization, Writing – original draft, Writing – review and editing.

## References

[B1] Abdi Z. Sharma T. (2004). Social cognition and its neural correlates in schizophrenia and autism. *CNS Spectr.* 9 335–343. 15115945 10.1017/s1092852900009317

[B2] Barak B. Feng G. (2016). Neurobiology of social behavior abnormalities in autism and Williams syndrome. *Nat. Neurosci.* 19 647–655. 29323671 10.1038/nn.4276 PMC4896837

[B3] Beier K. T. Kim C. K. Hoerbelt P. Hung L. W. Heifets B. D. DeLoach K. E. (2017). Rabies screen reveals GPe control of cocaine-triggered plasticity. *Nature* 549 345–350. 10.1038/nature23888 28902833 PMC6069680

[B4] Benekareddy M. Stachniak T. J. Bruns A. Knoflach F. von Kienlin M. Künnecke B. (2018). Identification of a corticohabenular circuit regulating socially directed behavior. *Biol. Psychiatry* 83 607–617. 10.1016/j.biopsych.2017.10.032 29336819

[B5] Benjamini Y. Hochberg Y. (1995). Controlling the false discovery rate: A practical and powerful approach to multiple testing. *J. R. Stat. Soc. Series B Stat. Methodol.* 57 289–300.

[B6] Bernaerts S. Boets B. Bosmans G. Steyaert J. Alaerts K. (2020). Behavioral effects of multiple-dose oxytocin treatment in autism: A randomized, placebo-controlled trial with long-term follow-up. *Mol. Autism* 11:6. 10.1186/s13229-020-0313-1 PMC6964112 31969977

[B7] Bicks L. K. Koike H. Akbarian S. Morishita H. (2015). Prefrontal cortex and social cognition in mouse and man. *Front. Psychol.* 6:1805. 10.3389/fpsyg.2015.01805 26635701 PMC4659895

[B8] Brownstein M. J. Russell J. T. Gainer H. (1980). Synthesis, transport, and release of posterior pituitary hormones. *Science* 207 373–378. 6153132 10.1126/science.6153132

[B9] Busnelli M. Kleinau G. Muttenthaler M. Stoev S. Manning M. Bibic L. (2016). Design and characterization of superpotent bivalent ligands targeting oxytocin receptor dimers via a channel-like structure. *J. Med. Chem.* 59 7152–7166. 10.1021/acs.jmedchem.6b00564 27420737

[B10] Caldwell H. K. (2018). Oxytocin and sex differences in behavior. *Curr. Opin. Behav. Sci.* 23 13–20.

[B11] Choe K. Y. Bethlehem R. A. I. Safrin M. Dong H. Salman E. Li Y. (2022). Oxytocin normalizes altered circuit connectivity for social rescue of the Cntnap2 knockout mouse. *Neuron* 110 795–808.e6. 10.1016/j.neuron.2021.11.031 34932941 PMC8944915

[B12] Contractor A. Ethell I. M. Portera-Cailliau C. (2021). Cortical interneurons in autism. *Nat. Neurosci.* 24 1648–1659. 34848882 10.1038/s41593-021-00967-6 PMC9798607

[B13] Dichter G. S. Felder J. N. Bodfish J. W. (2009). Autism is characterized by dorsal anterior cingulate hyperactivation during social target detection. *Soc. Cogn. Affect. Neurosci.* 4 215–226. 10.1093/scan/nsp017 19574440 PMC2728636

[B14] Dölen G. Darvishzadeh A. Huang K. W. Malenka R. C. (2013). Social reward requires coordinated activity of nucleus accumbens oxytocin and serotonin. *Nature* 501 179–184. 10.1038/nature12518 24025838 PMC4091761

[B15] Dumais K. M. Veenema A. H. (2016). Vasopressin and oxytocin receptor systems in the brain: Sex differences and sex-specific regulation of social behavior. *Front. Neuroendocrinol.* 40 1–23. 10.1016/j.yfrne.2015.04.003 25951955 PMC4633405

[B16] Ferguson B. R. Gao W.-J. (2018). PV interneurons: Critical regulators of E/I balance for prefrontal cortex-dependent behavior and psychiatric disorders. *Front. Neural Circuits* 12:37. 10.3389/fncir.2018.00037 29867371 PMC5964203

[B17] Forbes C. E. Grafman J. (2010). The role of the human prefrontal cortex in social cognition and moral judgment. *Annu. Rev. Neurosci.* 33 299–324. 20350167 10.1146/annurev-neuro-060909-153230

[B18] Foss-Feig J. H. Adkinson B. D. Ji J. L. Yang G. Srihari V. H. McPartland J. C. (2017). Searching for cross-diagnostic convergence: Neural mechanisms governing excitation and inhibition balance in schizophrenia and autism spectrum disorders. *Biol. Psychiatry* 81 848–861. 10.1016/j.biopsych.2017.03.005 28434615 PMC5436134

[B19] Grinevich V. Stoop R. (2018). Interplay between oxytocin and sensory systems in the orchestration of socio-emotional behaviors. *Neuron* 99 887–904. 10.1016/j.neuron.2018.07.016 30189208

[B20] Guastella A. J. Boulton K. A. Whitehouse A. J. O. Song Y. J. Thapa R. Gregory S. G. (2023). The effect of oxytocin nasal spray on social interaction in young children with autism: A randomized clinical trial. *Mol. Psychiatry* 28 834–842. 36302965 10.1038/s41380-022-01845-8 PMC9607840

[B21] Hara Y. Ago Y. Higuchi M. Hasebe S. Nakazawa T. Hashimoto H. (2017). Oxytocin attenuates deficits in social interaction but not recognition memory in a prenatal valproic acid-induced mouse model of autism. *Horm. Behav.* 96 130–136. 28942000 10.1016/j.yhbeh.2017.09.013

[B22] Hu B. Boyle C. A. Lei S. (2021). Activation of oxytocin receptors excites subicular neurons by multiple signaling and ionic mechanisms. *Cereb. Cortex* 31 2402–2415. 10.1093/cercor/bhaa363 33341872 PMC8023860

[B23] Huang Y. Huang X. Ebstein R. P. Yu R. (2021). Intranasal oxytocin in the treatment of autism spectrum disorders: A multilevel meta-analysis. *Neurosci. Biobehav. Rev.* 122 18–27. 10.1016/j.neubiorev.2020.12.028 33400920

[B24] Hung L. W. Neuner S. Polepalli J. S. Beier K. T. Wright M. Walsh J. J. (2017). Gating of social reward by oxytocin in the ventral tegmental area. *Science* 357 1406–1411. 28963257 10.1126/science.aan4994 PMC6214365

[B25] Jurek B. Neumann I. D. (2018). The oxytocin receptor: From intracellular signaling to behavior. *Physiol. Rev.* 98 1805–1908. 29897293 10.1152/physrev.00031.2017

[B26] Kehrer C. Maziashvili N. Dugladze T. Gloveli T. (2008). Altered excitatory-inhibitory balance in the NMDA-hypofunction model of schizophrenia. *Front. Mol. Neurosci.* 1:6. 10.3389/neuro.02.006.2008 18946539 PMC2525998

[B27] Kirchheim F. Tinnes S. Haas C. A. Stegen M. Wolfart J. (2013). Regulation of action potential delays via voltage-gated potassium Kv1.1 channels in dentate granule cells during hippocampal epilepsy. *Front. Cell. Neurosci.* 7:248. 10.3389/fncel.2013.00248 24367293 PMC3852106

[B28] Kitagawa K. Matsumura K. Baba M. Kondo M. Takemoto T. Nagayasu K. (2021). Intranasal oxytocin administration ameliorates social behavioral deficits in a POGZWT/Q1038R mouse model of autism spectrum disorder. *Mol. Brain* 14:56. 10.1186/s13041-021-00769-8 33726803 PMC7962304

[B29] Knobloch H. S. Charlet A. Hoffmann L. C. Eliava M. Khrulev S. Cetin A. H. (2012). Evoked axonal oxytocin release in the central amygdala attenuates fear response. *Neuron* 73 553–566. 10.1016/j.neuron.2011.11.030 22325206

[B30] Leng G. Leng R. I. Ludwig M. (2022). Oxytocin-a social peptide? Deconstructing the evidence. *Philos. Trans. R. Soc. Lond. B Biol. Sci.* 377:20210055. 10.1098/rstb.2021.0055 35858110 PMC9272144

[B31] Lewine J. D. Andrews R. Chez M. Patil A. A. Devinsky O. Smith M. (1999). Magnetoencephalographic patterns of epileptiform activity in children with regressive autism spectrum disorders. *Pediatrics* 104 405–418. 10469763 10.1542/peds.104.3.405

[B32] Lewis D. A. Hashimoto T. Volk D. W. (2005). Cortical inhibitory neurons and schizophrenia. *Nat. Rev. Neurosci.* 6 312–324. 15803162 10.1038/nrn1648

[B33] Li K. Nakajima M. Ibañez-Tallon I. Heintz N. (2016). A cortical circuit for sexually dimorphic oxytocin-dependent anxiety behaviors. *Cell* 167 60–72.e11. 10.1016/j.cell.2016.08.067 27641503 PMC5220951

[B34] Loomes R. Hull L. Mandy W. P. L. (2017). What is the male-to-female ratio in autism spectrum disorder? A systematic review and meta-analysis. *J. Am. Acad. Child Adolesc. Psychiatry* 56 466–474. 10.1016/j.jaac.2017.03.013 28545751

[B35] Markram K. Markram H. (2010). The intense world theory - a unifying theory of the neurobiology of autism. *Front. Hum. Neurosci.* 4:224. 10.3389/fnhum.2010.00224 21191475 PMC3010743

[B36] Marlin B. J. Mitre M. D’amour J. A. Chao M. V. Froemke R. C. (2015). Oxytocin enables maternal behaviour by balancing cortical inhibition. *Nature* 520 499–504. 25874674 10.1038/nature14402 PMC4409554

[B37] Meyer-Lindenberg A. Domes G. Kirsch P. Heinrichs M. (2011). Oxytocin and vasopressin in the human brain: Social neuropeptides for translational medicine. *Nat. Rev. Neurosci.* 12 524–538. 21852800 10.1038/nrn3044

[B38] Meziane H. Schaller F. Bauer S. Villard C. Matarazzo V. Riet F. (2015). An early postnatal oxytocin treatment prevents social and learning deficits in adult mice deficient for Magel2, a gene involved in prader-willi syndrome and autism. *Biol. Psychiatry* 78 85–94. 10.1016/j.biopsych.2014.11.010 25599930

[B39] Mubarik A. Tohid H. (2016). Frontal lobe alterations in schizophrenia: A review. *Trends Psychiatry Psychother.* 38 198–206. 28076640 10.1590/2237-6089-2015-0088

[B40] Murugan M. Jang H. J. Park M. Miller E. M. Cox J. Taliaferro J. P. (2017). Combined social and spatial coding in a descending projection from the prefrontal cortex. *Cell* 171 1663–1677.e16. 10.1016/j.cell.2017.11.002 29224779 PMC5889923

[B41] Nakajima M. Görlich A. Heintz N. (2014). Oxytocin modulates female sociosexual behavior through a specific class of prefrontal cortical interneurons. *Cell* 159 295–305. 10.1016/j.cell.2014.09.020 25303526 PMC4206218

[B42] Nelson S. B. Valakh V. (2015). Excitatory/inhibitory balance and circuit homeostasis in autism spectrum disorders. *Neuron* 87 684–698. 26291155 10.1016/j.neuron.2015.07.033 PMC4567857

[B43] Ongür D. Price J. L. (2000). The organization of networks within the orbital and medial prefrontal cortex of rats, monkeys and humans. *Cereb. Cortex* 10 206–219. 10.1093/cercor/10.3.206 10731217

[B44] Owen S. F. Tuncdemir S. N. Bader P. L. Tirko N. N. Fishell G. Tsien R. W. (2013). Oxytocin enhances hippocampal spike transmission by modulating fast-spiking interneurons. *Nature* 500 458–462. 10.1038/nature12330 23913275 PMC5283693

[B45] Pan L. Zheng L. Wu X. Zhu Z. Wang S. Lu Y. (2022). A short period of early life oxytocin treatment rescues social behavior dysfunction via suppression of hippocampal hyperactivity in male mice. *Mol. Psychiatry* 27 4157–4171. 10.1038/s41380-022-01692-7 35840800 PMC9718675

[B46] Peñagarikano O. (2017). Oxytocin in animal models of autism spectrum disorder. *Dev. Neurobiol.* 77 202–213. 27603327 10.1002/dneu.22449

[B47] Peñagarikano O. Lázaro M. T. Lu X.-H. Gordon A. Dong H. Lam H. A. (2015). Exogenous and evoked oxytocin restores social behavior in the Cntnap2 mouse model of autism. *Sci. Transl. Med.* 7:271ra8. 10.1126/scitranslmed.3010257 25609168 PMC4498455

[B48] Posserud M.-B. Skretting Solberg B. Engeland A. Haavik J. Klungsøyr K. (2021). Male to female ratios in autism spectrum disorders by age, intellectual disability and attention-deficit/hyperactivity disorder. *Acta Psychiatr. Scand.* 144 635–646. 34494265 10.1111/acps.13368

[B49] Rubenstein J. L. R. Merzenich M. M. (2003). Model of autism: Increased ratio of excitation/inhibition in key neural systems. *Genes Brain Behav.* 2 255–267. 14606691 10.1034/j.1601-183x.2003.00037.x PMC6748642

[B50] Schnider P. Bissantz C. Bruns A. Dolente C. Goetschi E. Jakob-Roetne R. (2020). Discovery of balovaptan, a vasopressin 1a receptor antagonist for the treatment of autism spectrum disorder. *J. Med. Chem.* 63 1511–1525. 10.1021/acs.jmedchem.9b01478 31951127

[B51] Sikich L. Kolevzon A. King B. H. McDougle C. J. Sanders K. B. Kim S.-J. (2021). Intranasal oxytocin in children and adolescents with autism spectrum disorder. *N. Engl. J. Med.* 385 1462–1473. 34644471 10.1056/NEJMoa2103583 PMC9701092

[B52] Song Z. Borland J. M. Larkin T. E. O’Malley M. Albers H. E. (2016). Activation of oxytocin receptors, but not arginine-vasopressin V1a receptors, in the ventral tegmental area of male Syrian hamsters is essential for the reward-like properties of social interactions. *Psychoneuroendocrinology* 74 164–172. 27632574 10.1016/j.psyneuen.2016.09.001 PMC6417503

[B53] Stegen M. Kirchheim F. Hanuschkin A. Staszewski O. Veh R. W. Wolfart J. (2012). Adaptive intrinsic plasticity in human dentate gyrus granule cells during temporal lobe epilepsy. *Cereb. Cortex* 22 2087–2101. 22038909 10.1093/cercor/bhr294

[B54] Stegen M. Young C. C. Haas C. A. Zentner J. Wolfart J. (2009). Increased leak conductance in dentate gyrus granule cells of temporal lobe epilepsy patients with Ammon’s horn sclerosis. *Epilepsia* 50 646–653. 10.1111/j.1528-1167.2009.02025.x 19292756

[B55] Stoop R. (2012). Neuromodulation by oxytocin and vasopressin. *Neuron* 76 142–159. 23040812 10.1016/j.neuron.2012.09.025

[B56] Suraev A. S. Bowen M. T. Ali S. O. Hicks C. Ramos L. McGregor I. S. (2014). Adolescent exposure to oxytocin, but not the selective oxytocin receptor agonist TGOT, increases social behavior and plasma oxytocin in adulthood. *Horm. Behav.* 65 488–496. 24631584 10.1016/j.yhbeh.2014.03.002

[B57] Tan Y. Singhal S. M. Harden S. W. Cahill K. M. Nguyen D.-T. M. Colon-Perez L. M. (2019). Oxytocin receptors are expressed by glutamatergic prefrontal cortical neurons that selectively modulate social recognition. *J. Neurosci.* 39 3249–3263. 10.1523/JNEUROSCI.2944-18.2019 30804095 PMC6788819

[B58] Triana-Del Río R. van den Burg E. Stoop R. Hegoburu C. (2019). Acute and long-lasting effects of oxytocin in cortico-limbic circuits: Consequences for fear recall and extinction. *Psychopharmacology* 236 339–354. 10.1007/s00213-018-5030-5 30302511

[B59] Turrigiano G. (2012). Homeostatic synaptic plasticity: Local and global mechanisms for stabilizing neuronal function. *Cold Spring Harb. Perspect. Biol.* 4:a005736. 10.1101/cshperspect.a005736 PMC3249629 22086977

[B60] Vattikuti S. Chow C. C. (2010). A computational model for cerebral cortical dysfunction in autism spectrum disorders. *Biol. Psychiatry* 67 672–678. 19880095 10.1016/j.biopsych.2009.09.008 PMC3104404

[B61] Viviani D. Charlet A. van den Burg E. Robinet C. Hurni N. Abatis M. (2011). Oxytocin selectively gates fear responses through distinct outputs from the central amygdala. *Science* 333 104–107. 10.1126/science.1201043 21719680

[B62] Voineagu I. Wang X. Johnston P. Lowe J. K. Tian Y. Horvath S. (2011). Transcriptomic analysis of autistic brain reveals convergent molecular pathology. *Nature* 474 380–384. 21614001 10.1038/nature10110 PMC3607626

[B63] Winter J. Meyer M. Berger I. Royer M. Bianchi M. Kuffner K. (2021). Chronic oxytocin-driven alternative splicing of Crfr2α induces anxiety. *Mol. Psychiatry* [Epub ahead of print]. 10.1038/s41380-021-01141-x 34035479 PMC10914602

[B64] Wise S. P. (2008). Forward frontal fields: Phylogeny and fundamental function. *Trends Neurosci.* 31 599–608. 10.1016/j.tins.2008.08.008 18835649 PMC2587508

[B65] Yizhar O. Levy D. R. (2021). The social dilemma: Prefrontal control of mammalian sociability. *Curr. Opin. Neurobiol.* 68 67–75. 10.1016/j.conb.2021.01.007 33549950

[B66] Yizhar O. Fenno L. E. Prigge M. Schneider F. Davidson T. J. O’Shea D. J. (2011). Neocortical excitation/inhibition balance in information processing and social dysfunction. *Nature* 477 171–178. 10.1038/nature10360 21796121 PMC4155501

[B67] Young C. C. Stegen M. Bernard R. Müller M. Bischofberger J. Veh R. W. (2009). Upregulation of inward rectifier K+ (Kir2) channels in dentate gyrus granule cells in temporal lobe epilepsy. *J. Physiol.* 587 4213–4233. 10.1113/jphysiol.2009.170746 19564397 PMC2754361

[B68] Young L. J. Barrett C. E. (2015). Neuroscience. Can oxytocin treat autism? *Science* 347 825–826. 25700501 10.1126/science.aaa8120 PMC4362686

[B69] Zhu W. Ding Z. Zhang Z. Wu X. Liu X. Zhang Y. (2021). Enhancement of oxytocin in the medial prefrontal cortex reverses behavioral deficits induced by repeated ketamine administration in mice. *Front. Neurosci.* 15:723064. 10.3389/fnins.2021.723064 34566567 PMC8462509

