# Supplementary material for: Selective oxytocin receptor activation prevents prefrontal circuit dysfunction and social behavioral alterations in response to chronic prefrontal cortex activation in male rats
Source: Front Cell Neurosci. 2023 Dec 7;17:1286552. doi: 10.3389/fncel.2023.1286552 (PMC10745491; doi:10.3389/fncel.2023.1286552)
Supplement: Supplementary file 1 [file Data_Sheet_1.docx]

**Supplementary material**

**Selective oxytocin receptor activation prevents prefrontal circuit dysfunction and social behavioral alterations in response to chronic prefrontal cortex activation in rats**

Names and affiliations of each author

Philipp Janz

Roche Pharma Research and Early Development, Neuroscience and Rare Diseases Discovery & Translational Area, Roche Innovation Center Basel, F. Hoffmann-La Roche AG, Grenzacherstrasse 124, 4070 Basel

Frederic Knoflach

Roche Pharma Research and Early Development, Neuroscience and Rare Diseases Discovery & Translational Area, Roche Innovation Center Basel, F. Hoffmann-La Roche AG, Grenzacherstrasse 124, 4070 Basel

Konrad Bleicher

Roche Pharma Research and Early Development, Therapeutic Modalities, Roche Innovation Center Basel, F. Hoffmann-La Roche AG, Grenzacherstrasse 124, 4070 Basel

Sara Belli

Roche Pharma Research and Early Development, Pharmaceutical Science, Roche Innovation Center Basel, F. Hoffmann-La Roche AG, Grenzacherstrasse 124, 4070 Basel

Barbara Biemans

Roche Pharma Research and Early Development, Neuroscience and Rare Diseases Discovery & Translational Area, Roche Innovation Center Basel, F. Hoffmann-La Roche AG, Grenzacherstrasse 124, 4070 Basel

Patrick Schnider

Roche Pharma Research and Early Development, Therapeutic Modalities, Roche Innovation Center Basel, F. Hoffmann-La Roche AG, Grenzacherstrasse 124, 4070 Basel

Martin Ebeling

Roche Pharma Research and Early Development, Pharmaceutical Science, Roche Innovation Center Basel, F. Hoffmann-La Roche AG, Grenzacherstrasse 124, 4070 Basel

Christophe Grundschober *

Roche Pharma Research and Early Development, Neuroscience and Rare Diseases Discovery & Translational Area, Roche Innovation Center Basel, F. Hoffmann-La Roche AG, Grenzacherstrasse 124, 4070 Basel

Madhurima Benekareddy *

Roche Pharma Research and Early Development, Neuroscience and Rare Diseases Discovery & Translational Area, Roche Innovation Center Basel, F. Hoffmann-La Roche AG, Grenzacherstrasse 124, 4070 Basel; Calico Life Science, 1170 Veterans Blvd., South San Francisco, CA 64080

(* co-last authors)

Corresponding author's name and email address

Name: Philipp Janz

Postal address: Roche Innovation Center Basel, Grenzacherstrasse 124, 4070 Basel, Switzerland

Email: [philipp.janz@roche.com](mailto:philipp.janz@roche.com)

Name: Madhurima Benekareddy

Postal address: Calico Life Science, 1170 Veterans Blvd., South San Francisco, CA 64080, USA

Email: [madhurimabr@gmail.com](mailto:madhurimabr@gmail.com)

**Supplementary Figures & Tables**


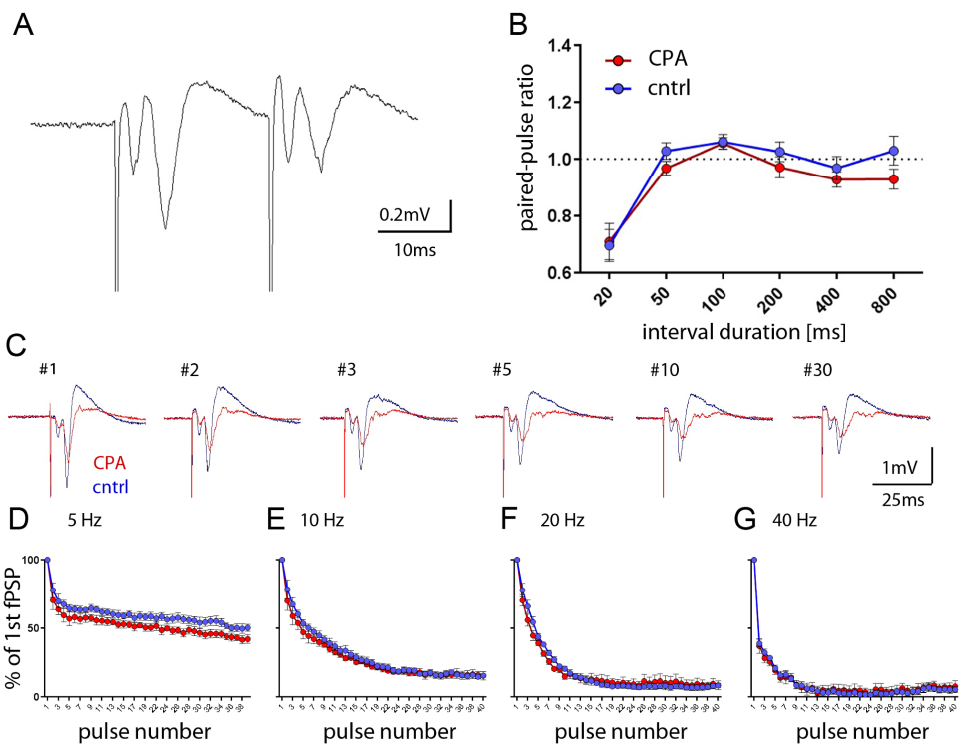


**Supplementary figure 1: Pre-pulse responses and short-term plasticity are preserved in PFC.** A) Representative voltage trace of a paired-pulse response at 20 ms inter-stimulus-interval duration. B) Quantitative analysis of paired-pulse ratios show no significant differences between groups. C) Representative evoked responses from a short-term depression protocol (here with 40 pulses presented at 5 Hz). D-G) Synaptic depression was quantified by inferring the %-change of the respective fPSP amplitude to the first fPSP. No significant differences are evident between groups for different stimulus frequencies (5, 10, 20 and 40 Hz). For paired-pulse and short-term depression protocols the stimulation intensity was used that elicited half maximum slope of the response (80-100 uA). Two-way ANOVA with Sidak`s post-hoc test was performed for statistical comparison. Data is displayed as mean ± SEM.


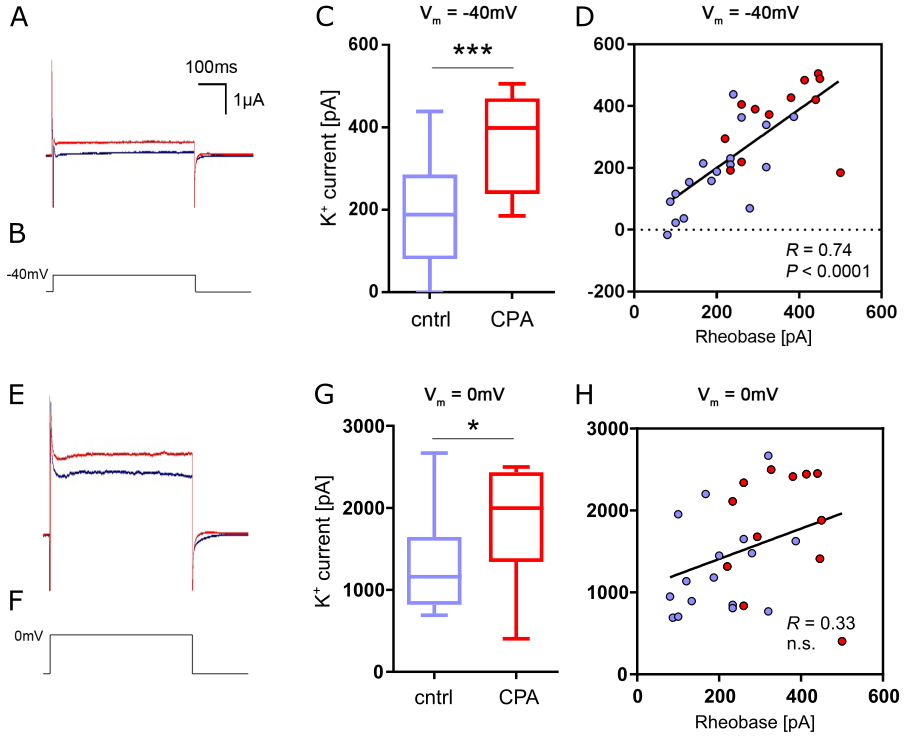


**Supplementary figure 2: Increased potassium currents.** A, B) Representative current traces at -40 mV membrane potential. Steady- state current represents the potassium current mediated by a subset of voltage-gated potassium channels. C) Quantitative analysis of the potassium steady-state current shows a highly significant increase for the CPA group. D) Linear regression analysis shows that this increase is positively correlated with the neuronal excitability represented by the rheobase. E-H) Same analysis performed for membrane potentials at 0 mV. Overall group comparison was performed with Student`s t-test. * p < 0.05, ** < 0.01, *** < 0.001. Linear regression analysis was done with Spearman’s correlation. Data is displayed as the median with min. to max. (box-and-whiskers).


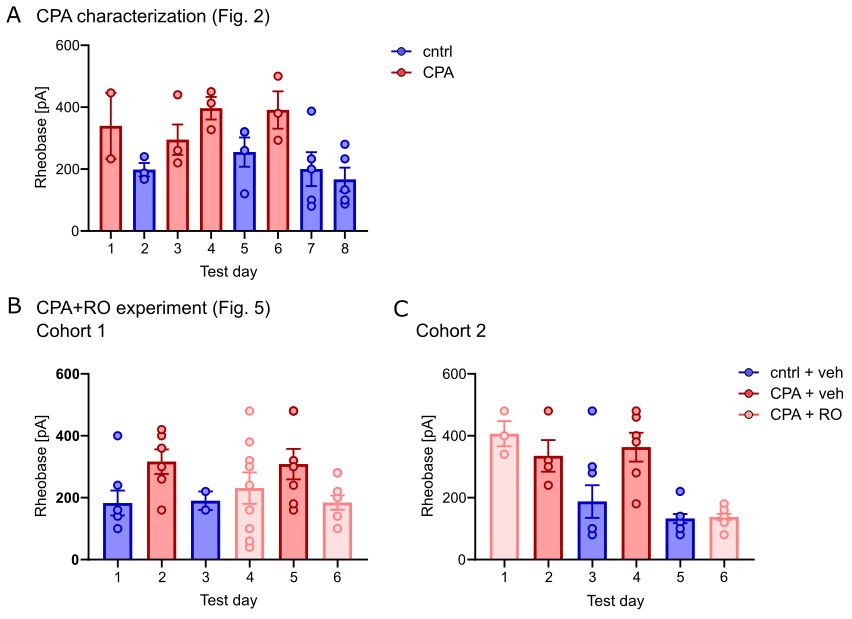


**Supplementary figure 3: Rheobase across cells and experiments plotted by individual test days.** A) Rheobase values as a readout of neuronal excitability for CPA characterization experiments. On each test day, acute slices were prepared from one either a control (blue) or a CPA rat (red). Individual data points refer to individual neurons patched in the respective animal. B, C) Same for cohort 1 and 2 from OXTR-activation experiments (RO = RO6958375). For each condition, individual cells show similar values across test days, indicating a stable phenotype.


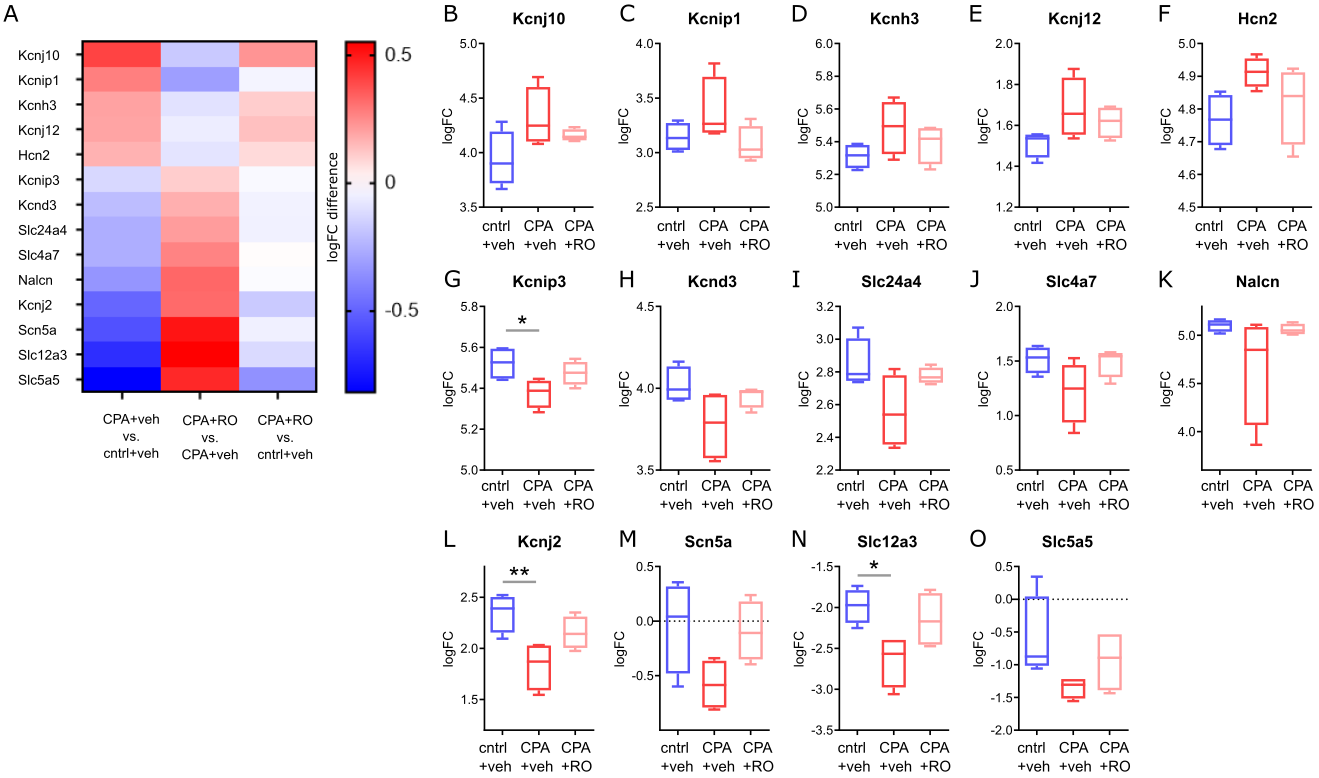


**Supplementary figure 4: Transcriptomic alterations focusing on ion channels involved in regulating cellular excitability.** A) Heatmap displaying significantly altered expression (between CPA+veh and control+veh, first column) of ion channels involved in cellular excitability. Countering of CPA-induced differential gene expression is evident followed by OXTR agonist (RO6958375, RO) treatment (second column), resulting in a normalization compared to control (third column).  B) Box plots for individual genes represented in the heatmap showing the fold change of gene expression in different conditions. Tested with one-way ANOVA and Tukey`s post-hoc test. * p < 0.05, ** < 0.01. Data is displayed as mean ± SEM.


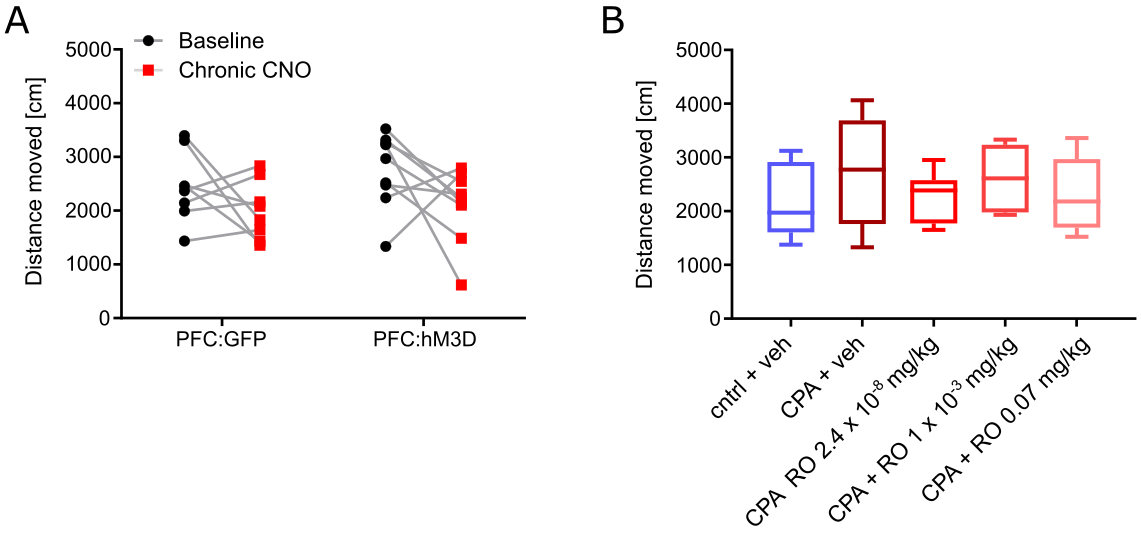


**Supplementary figure 5: Locomotor activity in 3-chamber sociability assays.** A) Total distance moved in sociability assay presented in figure 1C. Group comparison was performed using two-way ANOVA followed by Fisher’s LSD test. Group comparison was done using Sidak’s post-hoc test. B) Total distance moved in sociability assay presented in figure 5O. Data was analyzed using one-way ANOVA followed by Fisher’s LSD post-hoc test.


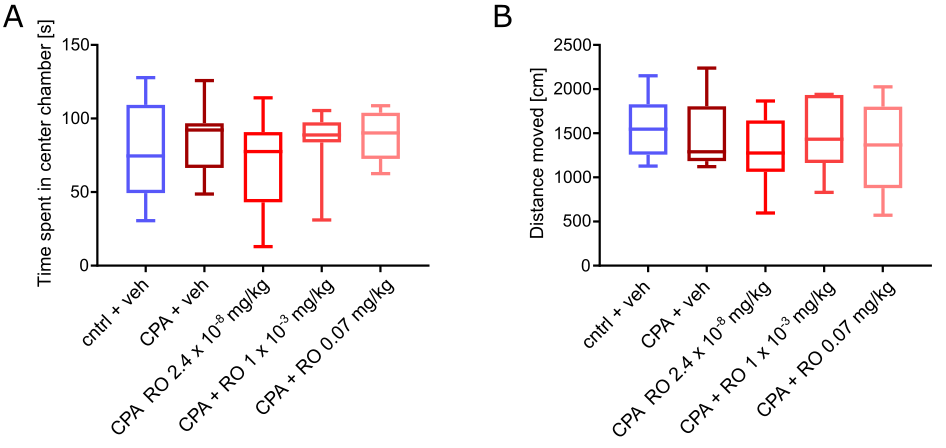


**Supplementary figure 6: Behavior in 3-chamber assays during the habituation phase.** A) Time spent in the center chamber and B) total distance moved in 3-chamber assay during the 10 minute habituation phase (no social or neutral object present). Data was analyzed using one-way ANOVA followed by Fisher’s LSD post-hoc test.

**Supplementary table 1: Numeric values and statistical tests for key results**. For each figure referenced in the results section mean 1 and mean 2 with standard error of the means (SEM) of the corresponding comparison is provided, including the p values and applied statistical test.

| **Figure** | **Comparison** | **Mean 1 ± SEM** | **Mean 2 ± SEM** | **p value** | **statistical test** |
| --- | --- | --- | --- | --- | --- |
| Fig. 1C | hM3Dq baseline vs. hM3Dq+CNO | 21.5 ± 4.1  (8 rats) | 9.6 ± 3.2  (8 rats) | 0.066 | 2-way ANOVA Fisher’s LSD |
|  | control+CNO vs. hM3Dq+CNO | 24.6 ± 4.5  (9 rats) | 9.6 ± 3.2  (8 rats) | < 0.05 | 2-way ANOVA Sidak’s test |
| Fig. 1H | AUC of control vs. AUC of CPA | 13.24 ± 0.82 mV  (4 rats) | 9.84 ± 0.65 mV  (5 rats) | < 0.05 | Unpaired Student’s t-test |
| Fig. 2B | control vs. CPA | -61.62 ± 1.97 mV  (21 cells, 4 rats) | -67.82 ± 1.89 mV  (17 cells, 4 rats) | < 0.05 | Unpaired Student’s t-test |
| Fig. 2C | control vs. CPA | 83.1 ± 6.36 MΩ  (21 cells, 4 rats) | 61.29 ± 6.89 MΩ  (17 cells, 4 rats) | < 0.05 | Unpaired Student’s t-test |
| Fig. 2H | AUC of control vs. AUC of CPA | 90.76 ± 18.48  (17 cells, 4 rats) | 17.24 ± 5.56  (12 cells, 4 rats) | < 0.01 | Unpaired Student’s t-test |
| Fig. 2I | control vs. CPA | 202.8 ± 22.4 mV  (17 cells, 4 rats) | 351.8 ± 28.2  (12 cells, 4 rats) | < 0.001 | Unpaired Student’s t-test |
| Fig. 2M | AUC of control vs AUC of CPA | 140 ± 15.41  (15 cells, 4 rats) | 75.14 ± 8.01  (12 cells, 4 rats) | < 0.01 | Unpaired Student’s t-test |
| Fig. 2N | control vs. CPA | 177 ± 24.55 MΩ  (16 cells, 4 rats) | 85.14 ± 8.5 MΩ  (12 cells, 4 rats) | < 0.01 | Unpaired Student’s t-test |
| Suppl. Fig. 2C | control vs. CPA | 187.6 ± 31.7 pA  (17 cells, 4 rats) | 365.8 ± 33.4 pA  (12 cells, 4 rats) | < 0.001 | Unpaired Student’s t-test |
| Suppl. Fig. 2G | control vs. CPA | 1313 ± 142.3 pA  (16 cells, 4 rats) | 1815 ± 201.7 pA  (12 cells, 4 rats) | < 0.05 | Unpaired Student’s t-test |
| Fig. 3D | control vs. CPA | 5.79 ± 1.43 Hz  (17 cells, 4 rats) | 9.90 ± 2.80 Hz  (14 cells, 4 rats) | 0.179 | Unpaired Student’s t-test |
| Fig. 3J | control vs. CPA | 1.21 ± 0.15 Hz  (19 cells, 4 rats) | 1.27 ± 0.15 Hz  (13 cells, 4 rats) | 0.784 | Unpaired Student’s t-test |
| Fig. 3K | control vs. CPA | 6.11 ± 1.1 Hz  (8 cells, 4 rats) | 4.58 ± 0.48 Hz  (5 cells, 4 rats) | 0.316 | Unpaired Student’s t-test |
| Fig. 5C | control+veh vs. CPA+veh | -63.66 ± 1.72 mV  (29 cells, 4 rats) | -72.17 ± 1.65 mV  (24 cells) | < 0.01 | 1-way ANOVA Tukey’s test |
|  | control+veh vs. CPA+RO | -63.66 ± 1.72 mV  (29 cells) | -66.87 ± 1.74 mV  (32 cells, 4 rats) | 0.364 | 1-way ANOVA Tukey’s test |
|  | CPA+veh vs. CPA+RO | -72.17 ± 1.65 mV  (24 cells, 4 rats) | -66.87 ± 1.74 mV  (32 cells, 4 rats) | 0.089 | 1-way ANOVA Tukey’s test |
| Fig. 5D | control+veh vs. CPA+veh | -82.38 ± 6.25 MΩ  (32 cells, 4 rats) | 63.6 ± 3.88 MΩ  (25 cells, 4 rats) | < 0.05 | 1-way ANOVA Tukey’s test |
|  | control+veh vs. CPA+RO | -82.38 ± 6.25 MΩ  (32 cells, 4 rats) | 77.59 ± 4.64 MΩ  (37 cells, 4 rats) | 0.774 | 1-way ANOVA Tukey’s test |
|  | CPA+veh vs. CPA+RO | 63.6 ± 3.88 MΩ  (25 cells, 4 rats) | 77.59 ± 4.64 MΩ  (37 cells, 4 rats) | 0.155 | 1-way ANOVA Tukey’s test |
| Fig. 5E | control+veh vs. CPA+veh | 61.31 ± 3.69 pF  (32 cells, 4 rats) | 79.54 ± 4.71 pF  (26 cells, 4 rats) | < 0.01 | 1-way ANOVA Tukey’s test |
|  | control+veh vs. CPA+RO | 61.31 ± 3.69 pF  (32 cells, 4 rats) | 65.11 ± 3.48 pF  (36 cells, 4 rats) | 0.754 | 1-way ANOVA Tukey’s test |
|  | CPA+veh vs. CPA+RO | 79.54 ± 4.71 pF  (26 cells, 4 rats) | 65.11 ± 3.48 pF  (36 cells, 4 rats) | < 0.05 | 1-way ANOVA Tukey’s test |
| Fig. 5K | control+veh vs. CPA+veh | 168.8 ± 20.59 mV  (25 cells, 4 rats) | 329.6 ± 22.56 mV  (23 cells, 4 rats) | < 0.001 | 1-way ANOVA Tukey’s test |
|  | control+veh vs. CPA+RO | 168.8 ± 20.59 mV  (25 cells, 4 rats) | 209 ± 22.38 mV  (29 cells, 4 rats) | 0.388 | 1-way ANOVA Tukey’s test |
|  | CPA+veh vs. CPA+RO | 329.6 ± 22.56 mV  (23 cells, 4 rats) | 209 ± 22.38 mV  (29 cells, 4 rats) | < 0.001 | 1-way ANOVA Tukey’s test |
| Fig. 5J | AUC of control+veh vs. AUC of CPA+veh | 16.56 ± 4.11  (25 cells, 4 rats) | 3.09 ± 1.65  (23 cells, 4 rats) | < 0.01 | 1-way ANOVA Tukey’s test |
|  | AUC of control+veh vs. AUC of CPA+RO | 16.56 ± 4.11  (25 cells, 4 rats) | 9.79 ± 2.74  (29 cells, 4 rats) | 0.249 | 1-way ANOVA Tukey’s test |
|  | AUC of CPA+veh vs. AUC of CPA+RO | 3.09 ± 1.65  (23 cells, 4 rats) | 9.79 ± 2.74  (29 cells, 4 rats) | 0.271 | 1-way ANOVA Tukey’s test |
| Fig. 5L | AUC of control+veh vs. AUC of CPA+veh | 38.64 ± 3.69  (29 cells, 4 rats) | 24.37 ± 1.89  (24 cells, 4 rats) | < 0.05 | 1-way ANOVA Tukey’s test |
|  | AUC of control+veh vs. AUC of CPA+RO | 38.64 ± 3.69  (29 cells, 4 rats) | 36.9 ± 4.56  (32 cells, 4 rats) | 0.940 | 1-way ANOVA Tukey’s test |
|  | AUC of CPA+veh vs. AUC of CPA+RO | 24.37 ± 1.89  (24 cells, 4 rats) | 36.9 ± 4.56  (32 cells, 4 rats) | 0.063 | 1-way ANOVA Tukey’s test |
| Fig. 5M | control+veh vs. CPA+veh | 198.6 ± 20.1 MΩ  (29 cells, 4 rats) | 119.8 ± 9.4 MΩ  (24 cells, 4 rats) | < 0.05 | 1-way ANOVA Tukey’s test |
|  | control+veh vs. CPA+RO | 198.6 ± 20.1 MΩ  (29 cells, 4 rats) | 180.7 ± 20.5 MΩ  (32 cells, 4 rats) | 0.758 | 1-way ANOVA Tukey’s test |
|  | CPA+veh vs. CPA+RO | 119.8 ± 9.4 MΩ  (24 cells, 4 rats) | 180.7 ± 20.5 MΩ  (32 cells, 4 rats) | 0.063 | 1-way ANOVA Tukey’s test |
| Fig. 5O | control+veh vs. CPA+veh | 188.14 ± 24.6 s  (8 rats) | 119.6 ± 13.3 s  (8 rats) | < 0.05 | 1-way ANOVA Fisher’s LSD |
|  | CPA+veh vs. CPA+RO 0.001mg/kg | 119.6 ± 13.3 s  (8 rats) | 198.6 ± 15.7 s  (8 rats) | < 0.05 | 1-way ANOVA Fisher’s LSD |
|  | CPA+veh vs. CPA+RO 0.07mg/kg | 119.6 ± 13.3 s  (8 rats) | 190.2 ± 23.6 s  (8 rats) | < 0.05 | 1-way ANOVA Fisher’s LSD |

**Supplementary table 2: EC50 values for selectivity tests measured in an in vitro Calcium release assay on cells expressing either rat or human receptors**. EC50 values are provided for RO6958375, oxytocin and vasopressin on either OXTR or V1aR showing that RO6958375 strongly activates OXTR but is inactive on V1aR.

| **Receptor** | **Compound** | **EC50** |
| --- | --- | --- |
| rat OXTR | RO6958375 | 0.011 ± 0.004 nM (8 experiments) |
|  | Oxytocin | 0.0048 ± 0.002 nM (7 experiments) |
| rat V1aR | RO6958375 | Inactive up to 27uM (7 experiments) |
|  | Oxytocin | 4.5 ± 0.8 nM (3 experiments) |
|  | Vasopressin | 0.011 ± 0.3 nM (3 experiments) |
| human OXTR | RO6958375 | 0.025 ± 0.004 nM (7 experiments) |
| human V1aR | RO6958375 | Inactive up to 27uM (7 experiments) |

**Supplementary table 3: Selectivity testing of the peptidic OXTR agonist RO6958375**. Testing was performed using radioligand binding and enzyme functional assays on 45 human receptors, channels and enzymes. Results showing an inhibition (or stimulation for assays run in basal conditions) higher than 50% are considered to represent significant effects of the test compounds. 50% is the most common cut-off value for further investigation Results showing an inhibition (or stimulation) lower than 20% compared to control values are not considered significant and mostly attributable to variability of the signal around the control level. Low to moderate negative values have no real meaning and are attributable to variability of the signal around the control level.

| **Receptor / channels transporter binding assays** | **% Inhibition of Control Specific Binding** | **1st / % of Control Specific Binding** | **2nd / % of Control Specific Binding** | **Mean / % of Control Specific Binding** | **Reference Compound** | **IC50 Ref [M]** | **Ki Ref [M]** |
| --- | --- | --- | --- | --- | --- | --- | --- |
|  |  |  |  |  |  |  |  |
| A1 | **-4** | 109.1 | 99.1 | 104.1 | CPA | 1.80E-09 | 7.20E-10 |
| A3 | **4** | 88 | 104.9 | 96.4 | IB-MECA | 2.70E-10 | 1.60E-10 |
| alpha 1A | **1** | 100.9 | 96.7 | 98.8 | WB 4101 | 2.90E-10 | 1.50E-10 |
| alpha 2A | **-7** | 107.6 | 105.8 | 106.7 | yohimbine | 4.70E-09 | 2.10E-09 |
| beta 1 | **-5** | 106.9 | 103.4 | 105.2 | atenolol | 2.50E-07 | 1.40E-07 |
| AT1 | **-1** | 96.1 | 106.6 | 101.4 | saralasin | 4.00E-10 | 2.00E-10 |
| BZD | **-16** | 115.9 | 116.2 | 116 | diazepam | 8.50E-09 | 7.10E-09 |
| D1 | **-2** | 117.6 | 86.8 | 102.2 | SCH 23390 | 4.20E-10 | 1.70E-10 |
| D2S | **6** | 94 | 93.4 | 93.7 | 7-OH-DPAT | 4.90E-09 | 2.00E-09 |
| glycine | **15** | 85.2 | 84.1 | 84.6 | glycine | 3.20E-07 | 2.90E-07 |
| H1 | **-1** | 102.3 | 99.5 | 100.9 | pyrilamine | 2.20E-09 | 1.40E-09 |
| H2 | **18** | 76.9 | 87 | 81.9 | cimetidine | 5.60E-07 | 5.40E-07 |
| H3 | **-6** | 112.6 | 99.3 | 105.9 | alpha-Me-histamine | 2.70E-09 | 6.60E-10 |
| I1 | **-6** | 94.4 | 116.7 | 105.5 | rilmenidine | 2.70E-07 | 1.40E-07 |
| M2 | **-14** | 119.6 | 108 | 113.8 | methoctramine | 6.90E-08 | 4.80E-08 |
| M4 | **-17** | 116.8 | 117 | 116.9 | 4-DAMP | 4.40E-10 | 2.70E-10 |
| N muscle-type | **5** | 93 | 96.3 | 94.7 | Alpha-bungarotoxin | 2.00E-09 | 1.80E-09 |
| kappa | **-13** | 115.4 | 110 | 112.7 | U 50488 | 1.20E-09 | 8.00E-10 |
| mu | **2** | 101.2 | 95.2 | 98.2 | DAMGO | 1.80E-09 | 7.40E-10 |
| PPARgamma | **-3** | 99.2 | 107 | 103.1 | rosiglitazone | 1.10E-08 | 5.90E-09 |
| PCP | **-6** | 116.2 | 94.8 | 105.5 | MK 801 | 4.70E-09 | 2.60E-09 |
| FP | **3** | 92.2 | 101.7 | 96.9 | PGF2alpha | 3.10E-09 | 2.00E-09 |
| 5-HT1A | **16** | 78.8 | 90 | 84.4 | 8-OH-DPAT | 8.60E-10 | 5.40E-10 |
| 5-HT2A | **9** | 88.6 | 92.6 | 90.6 | (±)DOI | 3.20E-10 | 2.40E-10 |
| 5-HT2B | **-2** | 103.2 | 100.7 | 102 | (±)DOI | 1.40E-08 | 7.00E-09 |
| 5-HT3 | **20** | 78.6 | 81.5 | 80 | MDL 72222 | 5.70E-09 | 4.00E-09 |
| sigma | **4** | 109.9 | 82.6 | 96.2 | haloperidol | 5.90E-08 | 4.80E-08 |
| sst4 | **17** | 85.8 | 79.9 | 82.8 | somatostatin-14 | 1.60E-09 | 1.60E-09 |
| GR | **-9** | 109.3 | 109 | 109.1 | dexamethasone | 2.90E-09 | 1.40E-09 |
| ERalpha | **-2** | 109.6 | 93.9 | 101.7 | 17-beta-estradiol | 5.30E-09 | 4.30E-09 |
| Ca2+ channel | **-36** | 129 | 143 | 136 | diltiazem | 5.40E-08 | 4.20E-08 |
| Na+ channel | **10** | 86.4 | 92.9 | 89.6 | veratridine | 1.10E-05 | 1.00E-05 |
| NE transporter | **3** | 98 | 96.4 | 97.2 | protriptyline | 2.60E-09 | 1.90E-09 |
| 5-HT transporter | **-4** | 106.7 | 100.3 | 103.5 | imipramine | 2.50E-09 | 1.20E-09 |
| **Enzyme functional assays** |  |  |  |  |  |  |  |
| COX2 | **15** | 87.2 | 83.3 | 85.3 | NS 398 | 5.80E-08 | 1.6 |
| PDE5 | **-21** | 130.8 | 112 | 121.4 | dipyridamole | 1.50E-06 | 1.4 |
| ACE | **-28** | 129.2 | 127.2 | 128.2 | captopril | 4.40E-10 | 1.6 |
| HIV-1 protease | **5** | 104.6 | 86.1 | 95.3 | pepstatin A | 1.70E-06 | 2.1 |
| CDK2 | **13** | 98.3 | 75.8 | 87.1 | staurosporine | 1.00E-08 | 1.1 |
| GSK3alpha | **-5** | 104.6 | 105.9 | 105.2 | staurosporine | 3.40E-08 | 1.8 |
| GSK3beta | **-2** | 106.3 | 97.8 | 102.1 | staurosporine | 5.20E-08 | 2 |
| acetylcholinesterase | **-2** | 101.1 | 101.9 | 101.5 | neostigmine | 4.30E-08 | 1.2 |
| MAO-A | **-15** | 123.1 | 107 | 115 | clorgyline | 3.90E-08 | 0.8 |
| MAO-B enzyme | **-14** | 114.1 | 113.6 | 113.8 | deprenyl | 3.30E-08 | 1.4 |
| xanthine oxidase / superoxide O2-scavenging | **8** | 96.1 | 88.2 | 92.1 | allopurinol | 5.60E-06 | 0.9 |
